# The *Lottia gigantea* shell matrix proteome: re-analysis including MaxQuant iBAQ quantitation and phosphoproteome analysis

**DOI:** 10.1186/1477-5956-12-28

**Published:** 2014-05-18

**Authors:** Karlheinz Mann, Eric Edsinger

**Affiliations:** 1 Max-Planck-Institut für Biochemie, Abteilung Proteomics und Signaltransduktion, Am Klopferspitz 18, Martinsried D-82152, Germany; 2 Rokhsar Department, Department of Molecular and Cell Biology, University of California, Berkeley, Berkeley, CA 94720, USA; 3 Present address: Brenner Unit, Okinawa institute of Science and Technology, 1919-0 Tancha, Onna-son, Kunigami-gun, Okinawa 904-0495, Japan

## Abstract

**Background:**

Although the importance of proteins of the biomineral organic matrix and their posttranslational modifications for biomineralization is generally recognized, the number of published matrix proteomes is still small. This is mostly due to the lack of comprehensive sequence databases, usually derived from genomic sequencing projects. However, in-depth mass spectrometry-based proteomic analysis, which critically depends on high-quality sequence databases, is a very fast tool to identify candidates for functional biomineral matrix proteins and their posttranslational modifications. Identification of such candidate proteins is facilitated by at least approximate quantitation of the identified proteins, because the most abundant ones may also be the most interesting candidates for further functional analysis.

**Results:**

Re-quantification of previously identified *Lottia* shell matrix proteins using the intensity-based absolute quantification (iBAQ) method as implemented in the MaxQuant identification and quantitation software showed that only 57 of the 382 accepted identifications constituted 98% of the total identified matrix proteome. This group of proteins did not contain obvious intracellular proteins, such as cytoskeletal components or ribosomal proteins, invariably identified as minor components of high-throughput biomineral matrix proteomes. Fourteen of these major proteins were phosphorylated to a variable extent. All together we identified 52 phospho sites in 20 of the 382 accepted proteins with high confidence.

**Conclusions:**

We show that iBAQ quantitation may be a useful tool to narrow down the group of functional biomineral matrix protein candidates for further research in cell biology, genetics or materials research. Knowledge of posttranslational modifications in these major proteins could be a valuable addition to previously published proteomes. This is true especially for phosphorylation, because this modification was already shown to modify mineralization processes in some instances.

## Introduction

Phosphorylation is one of the most widespread posttranslational modifications of proteins and also occurs in the organic matrix of biominerals [1,2]. Protein FAM20C has recently been identified as a kinase involved in phosphorylation of such secreted proteins [3,4], but other kinases may also be involved [5,6]. In a few cases experimental evidence indicated an important function for phospho groups in biomineral matrix proteins. The best-examined matrix phosphoprotein in this respect is mammalian osteopontin, first described as a major non-collagenous bone protein. Among the many functions suggested for this protein since its discovery (reviewed, for instance, in [7,8]) is also phosphorylation-dependent inhibition of mineralization processes [9]. Removal of phospho groups by alkaline phosphatase significantly reduces its inhibitory potential in *in vitro* crystallization assays [10] and un-phosphorylated recombinant osteopontin, but not *in vitro* phosphorylated osteopontin, fails to inhibit mineralization of human smooth muscle cell cultures serving as a model for human vascular calcification [11]. A crucial role of phosphorylated residues in the interaction with mineral is also reported for dentin matrix protein 1 and dentin phosphophoryn [12,13]. The only invertebrate example so far is orchestin, a major matrix protein from crustacean calcium storage structures. Phosphorylation of orchestin is necessary for calcium binding of the protein [14].

The recently published genomes of biomineralizing organisms enable high-throughput mass spectrometry-based analysis of biomineral proteomes and phosphoproteomes, thus facilitating the fast identification of phosphoproteins and phosphorylation sites [15,16]. In the present study we add the phosphoproteome of the *Lottia gigantea* shell matrix to the recently published *Lottia* shell proteomes [17,18]. Furthermore, we have re-quantitated the *Lottia* shell proteome using the iBAQ (intensity-based absolute quantification) method [19] as implemented in MaxQuant. This showed that 57 proteins make up 98% of the total identified proteome. We suggest that quantitation allows the identification of major proteins, which are the most likely candidates for functional shell proteins, while retaining information about minor proteins, irrespective of whether these minor proteins play a role in mineralization or not, and irrespective of whether they occur intra- or extra-crystalline.

## Materials and methods

### Matrix and phosphopeptide preparation

Lottia shell matrix was prepared as previously described [17] using method B for shell cleaning (2 h sodium hypochlorite incubation with 2 × 5 min ultrasound treatment). Reduction, carbamidomethylation and enzymatic cleavage of matrix proteins were performed using a modification of the FASP (Filter-aided sample preparation) method [20] as outlined below. Two-mg aliquots of acid-soluble or acid-insoluble shell matrix were suspended in 300 μl of 0.1 M Tris, pH8, containing 6 M guanidine hydrochloride and 0.01 M dithiothreitol (DTT). This mixture was heated to 56°C for 60 min, cooled to room temperature, and centrifuged at 13000 rpm in an Eppendorf bench-top centrifuge 5415D for 15 min. The supernatant was loaded into an Amicon Ultra 0.5 ml 30 K filter device (Millipore; Tullagreen, Ireland). DTT was removed by centrifugation at 13000 rpm for 15 min and washing with 2 × 1vol of the same buffer. Carbamidomethylation was done in the device using 0.1 M Tris buffer, pH8, containing 6 M-guanidine hydrochloride and 0.05 mM iodoacetamide and incubation for 45 min in the dark. Carbamidomethylated proteins were washed with 0.05 M ammonium hydrogen carbonate buffer, pH8, containing 2 M urea, and centrifugation as before. Trypsin (20 μg, Sequencing grade, modified; Promega, Madison, USA) was added in 40 μl of 0.05 M ammonium hydrogen carbonate buffer containing 2 M urea and the devices were incubated at 37°C for 16 h. Peptides were collected by centrifugation and the filters were washed twice with 40 μl of 0.05 M ammonium hydrogen carbonate buffer. The peptide solution was acidified to pH 1–2 with trifluoroacetic acid (TFA) and peptides were vacuum-dried in an Eppendorf concentrator.

Phosphopeptides were enriched by reversible binding to TiO_2_ beads (Titansphere 10 μm, GL Sciences, Japan) following established protocols [21] but substituting 2,5-dihydroxybenzoic acid in the loading buffer by 6% trifluoroacetic acid (TFA) [22]. Briefly, beads were washed first in 80% acetonitrile containing 0.1% TFA (washing buffer), then in 80% acetonitrile containing 6% TFA (binding buffer). Peptides were dissolved in binding buffer (200 μl/peptides of 2 mg matrix) and added to approximately 5 mg of loosely pelleted TiO_2_ beads. The mixture was incubated on a rotating wheel for 45 min. After centrifugation the supernatant was again incubated with fresh TiO_2_ beads as before. The beads were then washed twice with 200 μl of binding buffer followed by 2 × 200 μl of washing buffer. Finally the loaded beads were filled into C8 Stage Tips and phosphopeptides were eluted with 2 × 100 μl of a solution containing 40% acetonitrile and 15% ammonia. The eluate was vacuum-dried in an Eppendorf concentrator to ~20 μl and acidified with TFA. The peptides were purified on C18 Stage Tips [23] after dilution to 200 μl with 0.5% acetic acid.

### LC-MS analysis

Phosphopeptide-enriched samples were analysed on a Q Exactive high-performance Quadrupole Orbitrap mass spectrometer (Thermo Fisher Scientific, Bremen, Germany) [24] connected to an Easy-nLC 1000 nanoflow HPLC system (Thermo Fisher Scientific). Peptides were separated on a 50 cm column with an inner diameter of 75 μm filled with 1.8 μm C18 beads (Reprosil-AQ Pur, Dr. Maisch GmbH, Ammerbuch, Germany) prepared as described [25]. Peptides were eluted with acetonitrile in 0.1% formic acid using a gradient of 5-30% acetonitrile in 95min, 30-60% in 30 min and 60-95% in 8 min at a flow of 250 nl/min and a column temperature of 50°C [25]. Mass spectra were acquired in a data-dependent manner by automatically switching between MS and MS/MS in a top 10 approach. The resolution was 70000 for full spectra and 17500 (both at m/z 200) for HCD-derived fragments. The dynamic exclusion time was 30 sec.

### Data analysis

To estimate the percentage of each protein in the total identified shell proteome, raw-files used in a previous study [17; method B] were re-analysed using the iBAQ (intensity-based absolute quantification) method [19] as implemented in MaxQuant version 1.3.9.21. Carbamidomethylation was set as fixed modification, variable modifications were acetyl (protein N-term), oxidation (M), pyro-Glu (Q,E) and phospho (STY). Maximal FDR for peptide spectral match, proteins and site was set to 0.01. The maximal peptide PEP was 0.01. Minimal peptide length was 7 amino acids. The minimal score for modified peptides was 50 and the minimal delta score for modified peptides was 17. A minimum of two sequence-unique peptides was required for identification, except for proteins that were identified with two or more unique peptides previously in separately analysed acid-soluble and acid-insoluble fractions [17]. In very few cases new proteins were accepted with one unique peptide if this peptide occurred several times in different fractions and with an abundance of >0.01. The second peptide option was activated to enable identification of co-eluting peptides with very similar mass [26]. Two miss-cleavages were allowed. The databases used were Lottia FilteredModels (Lotgi1_GeneModels_FilteredModels1_aa.fasta.gz) and Lottia AllModels (Lotgi1_GeneModels_AllModels_20070424_aa.fasta.gz) [27] downloaded from (http://jgi.doe.gov/), and a LOTGI subset of UniProtKB v2013_7 entries downloaded from http://www.uniprot.org/. These were supplemented with the reversed sequences and common contaminants automatically and used for quality control and FDR setting by MaxQuant. Phosphopeptides were accepted if they occurred at least twice or were confirmed by analysis of phosphopeptide-enriched samples.

Peptide mixtures for enrichment of phosphopeptides were prepared from three biological replicates prepared according to method B of [17]. The acid-soluble and the acid-insoluble matrix of each biological replicate were used to prepare five technical replicates, resulting in 30 raw files that were evaluated together using MaxQuant [26,28] version 1.3.9.21 with the same settings as above with a minimum of one sequence-unique phosphopeptide only, but sequenced at least twice and in different replicates. The decoy mode was set to reward in MaxQuant. Phosphopeptide spectra were validated using the MaxQuant Expert system, which provides additional fragment annotations not included in the routine annotation [29]. Criteria were the assignment of major peaks, occurrence of uninterrupted y- or b-ion series of at least four consecutive amino acids, preferred cleavages N-terminal to proline bonds, the possible presence of a2/b2 ion pairs, the presence of immonium ions, and mass accuracy. In general only phosphopeptide identifications with a localization probability of ≥0.75 were accepted. However, in some cases adjacent residues, such as X_(n)_-S-S-X_(n)_, could not be resolved with the fragmentation pattern of the respective phosphopeptides, making it impossible to exactly localize the phosphorylation site. As a result, lower localization probability scores were attributed to several residues. Such phosphopeptides were also accepted. Phospho sites were searched for known kinase motifs using Phosida Motif Matcher (http://www.phosida.com/) [30,31] and PhosphoMotif Finder (http://www.hprd.org/PhosphoMotif_finder) [32]. Most sequence-unique peptides were identified several times and site occupancy of phospho sites was estimated by comparing the number of unmodified to the number of phosphorylated forms of individual peptides.

Sequence similarity searches were performed with FASTA (http://www.ebi.ac.uk/Tools/sss/fasta/) [33] against current releases of the Uniprot Knowledgebase (UniProtKB). Other bioinformatics tools used were Clustal Omega for sequence alignments (http://www.ebi.ac.uk/Tools/msa/clustalo/) [34], InterPro (http://www.ebi.ac.uk/interpro) [35] for domain predictions, and SignalP 4.1 (http://www.cbs.dtu.dk/services/SignalP/) [36] for signal sequence prediction. Amino acid composition and theoretical pI were determined using the ProtParam tool provided by the Expasy server (http://web.expasy.org/protparam/) [37]. Intrinsically disordered protein structure was predicted using IUPred (http://iupred.enzim.hu/) [38] and methods provided by the PredictProtein 2013 server (https://www.predictprotein.org/) [39,40]. GO categories for subcellular location were derived from UniProt and *Lottia* database entries, signal sequence predictions and similarity to known proteins.

## Results and discussion

### Re-analysis and re-quantitation of Lottia shell proteins with MaxQuant-implemented iBAQ

In search of the reasons for apparent differences in previously published *Lottia* shell proteomes [17,18] we noticed that database searches were done using the AllModels database in [18] while [17] used the FilteredModels database containing entries supported by EST sequences. Therefore we re-analyzed the raw-files produced previously for acid-soluble and acid-insoluble matrix prepared according to method B [17] (also used to identify phosphoproteins in the present report) using a combination of both databases and a subset of Uniprot containing Lottia + gigantea entries. Furthermore, to determine the approximate abundances of the identified proteins, the iBAQ (intensity-based absolute quantification) method [19] as implemented in more recent MaxQuant versions was enabled in this search. The previously used [17] emPAI method [41] belongs to the spectral count methods based on counting the number of identified unique parent ions per protein. In contrast, iBAQ and similar algorithms are called intensity-based because they calculate the sum of parent ion intensities of identified peptides per protein. In both types of methods, the numbers of theoretically possible peptides per protein for the protease used in sample preparation enter the equation to account for different protein lengths and distribution and frequency of cleavage sites. Comparison of the two different types of methods show a higher accuracy of the intensity-based methods, including iBAQ (for instance [42]), indicating that they should be given preference. Furthermore, the emPAI method in its original form [41] as we used it has become somewhat obsolete because of the recent progress in technology. For instance, modern mass spectrometers and the associated software provide high-confidence identifications of much longer peptides than previously possible. Consequently these long peptides are not included into emPAI calculations [41], but are included in iBAQ calculation.

Irrespective of the quantitation method accurate quantitation certainly also depends on the quality and completeness of the available sequence databases. Sequences not contained in the database can be neither identified by high-throughput mass spectrometry-based proteomic analysis nor quantitated. The same applies to sequences having no cleavage sites for the protease used in sample preparation. Faulty combination of sequences belonging to different proteins into one database entry or unnoticed faulty allocation of fragments of one protein to different database entries can all bias quantitation results. Finally, the abundance of proteins bearing many posttranslational modifications will be underestimated if the modification is not included in the analysis. In spite of these caveats we believe that routine quantitation of proteins in in-depth proteomic studies may be a useful tool to identify possible functionally important proteins for further study. We express the abundances as percentage of the identified proteome, obtained by normalizing the iBAQ intensities to the sum of all intensities. While the decision what to count as a major protein or a minor protein still remains arbitrary, it may now be more comprehensible to the reader and will possibly facilitate the decision of which proteins to choose for further studies.

The results of this new search (Additional file 1: Table S1) now includes all proteins published by [18] and contains 496 proteins/protein groups. Of these, 382 protein/protein group identifications were accepted (Additional file 2: Table S2) according to the rules stated in the Materials and Methods section. Twenty-three proteins were identified in the AllModels database only or in combination with the UniProt entries, including several very abundant ones (Table 1). Many groups contained several AllModels entries testifying to the high redundancy in this database. The corresponding MaxQuant table with protein data is contained in Additional file 1 (Additional file 1: Table S1), which also includes identifications not accepted. These were, for instance, identifications with only one single peptide with low scores or insufficient sequence coverage. The peptide data of the more than 4000 sequence-unique peptides, including peptide sequences and scores, are shown in Additional file 3 (Additional file 3: Table S3).

**Table 1:** Fifty-seven proteins with an individual percentage of equal to or larger than 0.1% constitute 98% of the total identified proteome

**Protein**	**Accession-no.**	**% of total identified proteome**	**Phospho-rylation**
**Aspartate-, glycine-, lysine- and serine-rich protein/B3A0P1/peroxidase-like protein 1**; domain: haem_peroxidase (~aa40-675); 20% G, 12% S; pI 4.96; GO: extracellular; DS: most of aa680-1870	Lotgi1|162078 DGLSP_LOTGI^2^	16.71	++
**Proline-rich protein 1/B3A0Q1**; 11% A, 13% P; pI:9.72; GO: extracellular; DS: C-terminal 15aa	Lotgi1|235497^1^ PRP1_LOTGI^2^	12.28	(+)
**Glycine- and methionine-rich protein/B3A0R1**; 12% A, 20% G, 10% L, 18% M, pI:11.24; GO: extracellular; DS: aa125-225	Lotgi1|239174^1^ GMP_LOTGI^2^	9.14	
**Glycine- and Serine rich protein-1**/B3A0P6; 10% A, 20% G, 13% S; pI 9.0; GO: extracellular; DS: ~aa67-84 (18aa)	Lotgi1|239214 GSP1_LOTGI^2^	6.82	(+)
**Peroxidase-like protein 2**/B3A0P3; domains: haem_peroxi-dase (~aa666-1124); 13% G, 11% S; pI 8.52; GO: extracellular; DS: ~aa1-620, aa1197-1492	Lotgi1|232817^1^ PLSP2_LOTGI^2^	6.80	++
**Glycine-rich protein**/B3A0R2; 10% A, 16% G, 12% M, 10% L; pI 9.87; GO: extracellular; DS: aa127-145 (19aa)	Lotgi1|239170^1^ GRP_LOTGI^2^	5.91	
**Uncharacterized shell protein 5**/B3A0Q0; 13% A, 11% R, 11% L; pI 10.32; GO: extracellular; DS: short stretches especially in C-terminal half	Lotgi1|238831^1^ USP5_LOTGI^2^	5.11	
**Coiled-coil domain-containing protein 1**/B3A0Q3; domain: coil; 31% D; pI 3.55; GO: extracellular; DS: short stretches all over aa27-394	Lotgi1|233420^1^ CCD1_LOTGI^2^	3.49	++
**Similar to blue mussel shell protein (BMSP)/**similar to collagen α4 (VI); domains: VWA; 11% I; pI 8.33; GO: extracellular; DS: none	Lotgi1|140660^1^ Lotgi1|173139^2^	2.81	
**Uncharacterized shell protein 13**/B3A0R3; 10% G; pI 8.32; GO: extracellular; DS: ~aa180-291	Lotgi1|234885^1^ USP13_LOTGI^2^	2.13	
**Uncharacterized shell protein 16**/B3A0R5; pI 9.63; GO: extracellular; DS: none	Lotgi1|231046^1^ USP16_LOTGI^2^	2.01	
**Proline-rich protein 2**/B3A0R8; 16% P; pI 9.98; GO: extracellular; DS: short stretches especially in aa161-186	Lotgi1|230510^1^ PRP2_LOTGI^2^	1.67	
**Glycine-, glutamate-and proline-rich protein**/B3A0P5; domain: Lysozyme_like (~aa240-415); 12% Gly; pI 5.08; GO: extracellular; DS: aa73-137, aa201-218	Lotgi1|231311^1^ GEPRP_LOTGI^2^	1.45	+
**Methionine-rich protein**/B3A0R7; 10% N, 11% P; pI 9.62; GO: extracellular; DS: ~aa50-400	Lotgi1|173200^1^ MRP_LOTGI^2^	1.43	
**Uncharacterized shell protein 26**/B3A0P4/BMSP-like; 18% G, 12% S, 10% T; pI 9.11; GO: extracellular; DS: small segments scattered over entire sequence	Lotgi1|238526^1^ USP26_LOTGI^2^	1.42	+
**Uncharacterized shell protein 8**/B3A0Q4; 11% P, 10% Y; pI 9.71; GO: extracellular; DS: short regions interspersed throughout the sequence	Lotgi1|228268^1^ USP8_LOTGI^2^	1.22	+
**Uncharacterized protein**; 10% Q (C-term), 11% P; pI 9.67; GO: none; DS: : small segments scattered over entire sequence	Lotgi1|158113^1^	1.19	
**Uncharacterized/similar to superoxide dismutase**; domain: SOD; 12% P, 10% Q; pI 9.30; GO: intracellular/extracellular; DS: ~aa20-450; SOD:~aa480-635	Lotgi1|166131 Lotgi1|101611^1^	1.09	+
**SCP domain-containing protein 2**/B3A0P8; domain: CAP (~aa145-310); pI 9.56; GO: extracellular; DS: ~aa16-155	Lotgi1|233200^1^ SCP2_LOTGI^2^	0.97	
**Similar to nacrein-like protein**/putative carbonic anhydrase 1/B3A0P2; domain: α-carbonic anhydrase; pI 6.44; GO: extracellular; DS: none	Lotgi1|238082^1^ CAH1_LOTGI^2^	0.96	
**Putative carbonic anhydrase 2**; aa190-632 100% identity to CAH2/B3A0Q6; domain: α-CA (~aa85-411); 11% R, 13% D, 13% G; pI 5.87; GO: extracellular; DS: aa415-633	Lotgi1|239188^1^ CAH2_LOTGI^2^	0.88	
**Uncharacterized protein**; 10% A, 12% L; pI 9.77; GO: extracellular; DS: few to none	Lotgi1|231009^1^	0.87	
**Uncharacterized protein**; domain: CBM_14 (chitin-binding)/peritrophin A (~aa18-87); pI 6.65; GO: extracellular; DS: none	Lotgi1|173138^1,2^	0.87	
**Uncharacterized protein**; domain: IGFBP_Nterm; 11% C, 10% S; pI 9.03; GO: extracellular; DS: none	Lotgi1|174065^1^	0.81	
**Uncharacterized shell protein 4**/B3A0P9; 10% S, 12% Y; pI 8.89; Go: extracellular; DS: possibly short C-tern segment	Lotgi1|236183^1^ USP4_LOTGI^2^	0.77	
**Glycine and tyrosine-rich protein**/B3A0Q2; 14% G, 13% T; pI 5.43; GO: extracellular; DS: most of the sequence	Lotgi1|235621^1^ GTRP_LOTGI^2^	0.71	
aa151-448 96% identity to **coiled-coil domain-containing protein 2**/B3A0Q7; 10% D, 20% G (GM/GGG-rich C-terminus (~aa430-630); pI 3.77; GO: extracellular; DS: most of aa290-410	Lotgi1|234936 CCD2_LOTGI^2^	0.67	
**Uncharacterized protein**; domains: antistasin, WAP; 16%C,11% P, pI 5.62; GO: extracellular; DS: none	Lotgi1|239125^1^ Lotgi1|226725	0.66	
**Uncharacterized protein/glycosidase 2**; domain: DUF187; similar to GEPRP_LOTIA (37% identity); pI 4.76; GO: extracellular; DS: ~aa78-130	Lotgi1|174920^1,2^	0.64	+
**Uncharacterized protein**/similar to ER aminopeptidase; domain: peptidase_M1, ERAP1_LIKE_C; pI 8.94; GO: ER/Golgi/ext. plasma membrane; DS: none	Lotgi1|140786^1^ Lotgi1|225855	0.61	
**SCP domain-containing protein 1**/B3A0P7; domain: CAP (aa143-305); 11% S; pI 9.21; GO: extracellular; DS: ~aa20-110	Lotgi1|233199^1^ SCP1_LOTGI^2^	0.53	
**Uncharacterized Gly-rich protein**; 12% N, 22% G; pI 9.54/9.30; GO: extracellular; DS: ~aa40-200 (275200)	Lotgi1|239447^1^ Lotgi1|175200	0.47	
**Similar to chorionic proteinase inhibitor/perlwapin-like**; domains: WAP (5x); aa1-125 99.6% identity to B3A0S1; 11% C, 10% P; pI 7.84; GO: extracellular; DS: none	Lotgi1|239121 Lotgi1|201802 PWAPL_LOTGI^2^	0.39	
**Uncharacterized protein**; pI 9.49; GO: none; DS: none	Lotgi1|234387^1^	0.38	
**Proline-rich protein 3**/B3A0S4; 10% N, 11% G, 13% P; pI 9.56; GO: extracellular; DS: few short segments	Lotgi1|237996^1^ Lotgi1|172116 PRP3_LOTGI^2^	0.34	
**EGF-like domain-containing protein 1** (aa170-682 of entry)/B3A0R6; domains: EGF (aa241-277), zona _pellucida (ZP; aa284-534); pI 5.80; GO: extracellular; DS: ~aa525-620	Lotgi1|235548^1^ ELDP1_LOTIA^2^	0.27	
**Peroxidase-3**/B3A0Q8; domain: haem_peroxidase (aa531-1077); 13% N; pI 7.5; GO: extracellular; DS: 26-381	Lotgi1|232818^1^ Lotgi1|99809 PLSP3_LOTGI^2^	0.26	
**Uncharacterized protein**/LUSP_10; 16% A, 17% D; pI 3.82; GO: extracellular; DS: most of the sequence	Lotgi1|163637^1,2^	0.25	
**Uncharacterized protein**; Pro/Ala- and His-rich motifs in C-term; pI 8.78; GO: extracellular; DS: short segments scattered over entire sequence	Lotgi1|233397^1^ Lotgi1|163339	0.24	
**Similar to peptidyl-prolyl cis/trans isomerase**/B3A0R0; domain: cyclophilin_type_PPI; 13% G; pI 4.75; GO: extracellular; DS: none	Lotgi1|222979^1^ Lotgi1|169679 PPI_LOTGI^2^	0.24	
**Uncharacterized**; domains: VWC/pacifastin; 13% C, 12% D, 11% S; pI 3.87; GO: extracellular; DS: none	Lotgi1|230854^1^ Lotgi1|99757	0.23	
**Uncharacterized Gln-rich protein**; 26% Q, 13% L, 12% T; pI 4.02; GO: extracellular; DS: ~aa40-320	Lotgi1|159331^1^	0.22	
**Uncharacterized Pro-rich protein**; 15% P; pI 9.50: GO: extracellular; DS: aa32-416	Lotgi1|174003^1^	0.22	
**Uncharacterized protein**/LUSP-18; 15% P, 15% T; pI 5.73; GO: extracellular; DS: ~aa18-557	Lotgi1|235610^1,2^	0.20	
**EGF-like domain_containing protein 2**/B3A0S3; domains: EGF (aa73-109), ZP (aa116-370); pI 4.9; GO: extracellular; DS: few (aa364-386,403-425)	Lotgi1|167423 ELDP2_LOTGI^2^	0.19	
**Uncharacterized protein**/Similar to PIF; 41% identity to PIF_PINFU aa427-526; domain: ConA_like_lectin; pI 8.91; GO: extracellular; DS: none	Lotgi1|237510^1^ Lotgi1|171086	0.16	
**Uncharacterized protein**/LUSP-14; domain: chitin_binding_3; pI 8.77; GO: extracellular; DS: aa225-251	Lotgi1|226726^1^ Lotgi1|239129^2^	0.16	+
**Uncharacterized protein**; 28% identical to PIF_PINFU: domains: VWA, chitin-binding, ConA_like_lectin; pI 5.15; Go: extracellular; DS: none	Lotgi1|228264^1^	0.15	
**Uncharacterized Gln-rich protein**; domain: Sushi/SCR/CCP (aa158-212); 19% Q, 11% P; pI 9.19; GO: extracellular; DS: most of the sequence	Lotgi1|234884^1^ Lotgi1|166202	0.14	
**Uncharacterized protein**; aa1-138 100% identity to ASRP/B3A0S2; 10% A, 10% N, 19% D, 11% V; pI 3.73 acid C-term half); GO: extracellular; DS: aa43-232	Lotgi1|238358^1^ ASRP_LOTGI^2^	0.14	+
**Uncharacterized protein**; 13% S; pI 4.43; GO: extracellular; DS: aa47-338	Lotgi1|171084^1^	0.11	+
**Perlustrin-like**/B3A0Q9; 43% identity to PLS_HALLA; domain: IGFBP_N; 11% C, 11% E; pI 4.05; GO: extracellular; DS: none	Lotgi1|238970^1^ PLSLP_LOTGI^2^	0.11	
**Uncharacterized protein**; 10% Q, 10% P, 11% S; pI 9.79; GO: extracellular; DS:~aa90-928	Lotgi1|158316^1^	0.10	
**Uncharacterized protein**; domain: SOUL_haem_binding; 13% L; pI 6.96; GO: extracellular; DS: none	Lotgi1|205030^1^ Lotgi1|237594	0.10	
**Uncharacterized protein**; 11% E, pI 4.32; GO: none (transmembrane?); DS: aa426-669 and smaller segments	Lotgi1|154020^1^	0.10	++
**Uncharacterized shell protein 22**/B3A0S0; 21% Q, 18% P; pI 8.43; GO: extracellular; DS: most of the sequence	Lotgi1|236690^1^ USP22_LOTGI^2^	0.10	
**Uncharacterized protein/LUSP-20**; domains: chitin_binding CBM_14/peritrophin A (aa384-504); 13% T; pI 6.79; GO: extracellular; DS: most of ~ aa60-380	Lotgi1|239574^1,2^	0.10	

+, less than three peptides phosphorylated; ++, three or more phosphopeptides; (+), not confirmed with phosphopeptide-enriched samples. DS, predicted disordered structure. ^1^, previously identified by Mann et al., 2012 [17]; ^2^, previously identified by Marie et al., 2013 [18].

**Figure 1:**
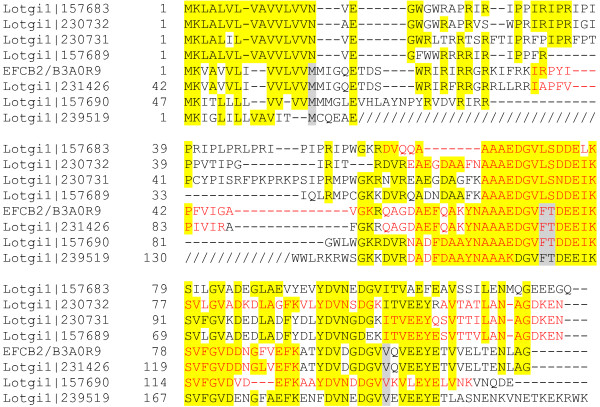
**Alignment of EFCB2 to similar sequences.** Sequences covered by MS/MS-sequenced peptides are shown in red. Slashes in the sequence of Lotgi1|239519 indicate an insert between signal peptide and the EFCB2-like sequence that does not occur in the other entries. All shown entries were part of protein groups containing other similar sequences due to the high redundancy of the AllModels database.

Quantitation with iBAQ showed that only 18 proteins/protein groups of a percentage of more than 1% of the identified proteome already constituted approximately 82% of the entire identified proteome (Table 1). This group comprised two very abundant (>1%) proteins not contained in the FilteredModels database, the Asp-, Gly-, Lys- and Ser-rich peroxidase-like protein-1 (DGLSP_LOTGI/Lotgi1|162078) and the Gly- and Ser-rich protein-1 (GSP1_LOTGI/Lotgi1|239214) [18]. If a percentage of larger than 0.1% was chosen as a threshold, a total of 57 proteins (Table 1) amounted to approximately 98% of the total identified proteome. These included CCD2 (coiled-coil domain-containing protein 2; Lotgi1|234936), the perlwapin-like protein PWAP_LOTGI/Lotgi1|239121, and the EGF-like domain-containing protein 2 (ELDP2/Lotgi1|167423) [15], which were contained in the AllModels database but not in the FilteredModels database. Almost all proteins also identified in [18] were contained in this fraction of the proteome. Exceptions were the EF-hand calcium-binding domain-containing protein 1 and 2 (EFCB1/B3A0Q5, EFCB2/B3A0R9), and Threonine-rich protein LUSP-15/TRP/B3A0R4, which apparently belonged to the minor components of the identified proteome (Additional file 2: Table S2). However, we also identified several entries with a high similarity to EFCB2 based on sequence overlaps with sequence identities of 43-90% (Figure 1). Taken together, this protein family constituted slightly more than 0.1% of the identified proteome.

In agreement with a previous study [18] the major proteins comprised three peroxidase-like proteins (Table 1) including the most abundant protein Lotgi|162078/DGLSP_LOTGI. Peroxidases are a large and widespread family of enzymes catalysing redox reactions using a variety of electron donors and acceptors, including organic molecules. Peroxidases have been implicated previously in mollusc shell formation [43]. Possibly they are responsible for the sclerotization of the periostracum [44-46], a proteinaceous layer confining the mantle cavity before the start of mineralization. As discussed previously [18] one may hypothesize that peroxidases function in stabilization of the newly secreted matrix by cross-linking some of its components. Another major protein, the abundance of which was noticed only using the AllModels database because the FilteredModels only contained a small fragment, was Lotgi1|166131. In this protein a long stretch of sequence with predicted disordered structure is followed by a predicted superoxide dismutase domain. Superoxide dismutases are a family of enzymes with widespread subcellular distribution that remove superoxide, a normal aerobic metabolite. One reaction product of superoxide dismutases is H_2_O_2_, a substrate of peroxidases.

In general, very little is known about the possible functions of shell matrix proteins, but in some cases similarities to known proteins and predicted domain structures may provide some clues for further studies. Predicted domain structures, GO terms for subcellular location, unusual amino acid composition features (amino acids representing ≥ 10% of the sequence) and theoretical isoelectric point for major identified Lotgi entries are included in Table 1. Extremely acidic matrix proteins (pI below 4.5) have found much interest in biomineralization research because of the possibility of direct interaction with the positively charged biomineral cations and have been hypothesized to act as nucleation sites involved in crystal formation [47]. The group of 57 proteins with an abundance of >0.1 includes eight of such uncharacterized unusually acid proteins (Table 1) that may deserve to be studied in more detail. Many proteins isolated from biominerals contain sequence regions of intrinsically disordered structure, a feature that is implicated in protein-protein interaction and mineral binding [48,49]. Table 1 includes several proteins with extended sequence regions of predicted disordered structure, such as the peroxidase-like protein-1 (DGLSP_LOTGI), the methionine-rich protein MRP_LOTGI, peroxidase_like 3 (PLSP3_LOTGI), and the uncharacterized proteins in Lotgi1|163637, 159331, 235610, 234884, 171084, 158316, 236690, and 239574. In two sequences both features, unusual acidity and predicted long-range structural disorder, coincide (Lotgi|159331, 171084). However, like all predicted features, predicted structural disorder needs experimental validation before far-reaching conclusions can be drawn.

Sometimes predicted domains strongly indicate involvement of the respective protein in biomineralization events. The putative carbonic anhydrases encoded in Lotgi|238082/CAH1 and Lotgi|239188/CAH2 and discussed previously [18] may be important for carbonate ion delivery. Also of special interest are proteins containing chitin-binding domains, such as Lotgi1|226726, 228264, and 239574. Many mollusc shells contain chitin-based extra-crystalline scaffolds and chitin-binding proteins may be important for organizing such scaffolds or may mediate interactions between chitin and the calcified matrix [50]. However, for most proven and putative shell matrix proteins the function remains unknown at present.

Most of the identified proteins were only minor, or trace, components that may not have a function in biomineralization. However, it should be emphasised that there may be exceptions. For example, protein FAM20C (0.006% of the *Lottia* shell proteome; Additional file 2: Table S2), was recently identified as a Golgi apparatus kinase responsible for the phosphorylation of many secreted proteins, including proteins important for biomineralization [3,4]. This kinase is also secreted to some degree, may be active in the extracellular space [5], and may enter biominerals in the company of its substrates. Of course this does not imply any function within the matrix but may explain its presence there. Other examples of the possible importance of trace components for biomineral formation are the sea urchin spicule proteins P58-A and P58-B. The extracellular domains of these predicted transmembrane proteins were detected as minor components in sea urchin spicule matrix [51] and both were subsequently shown by knock-down experiments to play an essential role in sea urchin larval skeletogenesis [52]. Also among the trace components are proteins known to have a predominantly intracellular location, such as cytoskeletal components and cytosolic enzymes (Additional file 2: Table S2). We think that these proteins do not have a function in biomineralization. However, even trace components with a well-defined intracellular role, such as ubiquitin (now also known to occur in the extracellular space, however [53]) may have a true role in biomineralization, such as in the matrix of the *Pinctada fucata* shell prismatic layer [54]. Finally it should be considered that the number of up-regulated genes, for instance after shell damage [55], is usually much larger than the number of major proteins identified in shell matrices. Possibly many of the trace proteins reflect regulatory or catalytic processes involved in the mineralization event at some point.

### The phosphoproteome

Because of the low number of different proteins in the shell matrix and because the HCD (higher energy collisional dissociation) fragmentation method used in the previous shell proteome analysis [17] enables phosphopeptide analysis at high resolution and mass accuracy in the LTQ Orbitrap Velos [56,57] without the need for neutral loss-dependent MS^3^ or multistage activation [58] used previously with CID fragmentation, we included phosphorylation as a variable modification in this re-analysis. The results indicated (Additional file 1: Table S1) that several major and a few minor proteins were phosphorylated to a variable extent. These preliminary results were validated by analysis of phosphopeptide-enriched samples of shell matrix proteins (Additional file 4: Table S4). Thirteen of these were confirmed by analyzing phosphopeptide-enriched fractions. Three more were identified only in phosphopeptide-enriched samples (Additional file 4: Table S4), yielding a total of 20 phosphoproteins. The MaxQuant phosphopeptide output table is shown in Additional file 5: Table S5. Nine major proteins with a percentage of more than 1% of the identified protein and five with a percentage between 0.1% and 1% (Table 1) were identified as phosphoproteins. Simultaneous determination of phosphorylated and non-phosphorylated versions of the phosphopeptides in the general survey without prior enrichment enabled an approximate estimation of site occupancy (Additional file 4: Table S4), which was very low in most cases. Site occupancy in the group of major proteins was highest in GEPRP/B3A0P5 and the uncharacterized protein of Lotgi1|154020. While GEPRP contained only two closely spaced phosphorylation sites, Lotgi1|154020 contained four sites in three peptides (Additional file 4: Table S4). This high site-occupancy strongly indicates that phosphorylation of these proteins may be functionally important. Three proteins, DGLSP/B3A0P1, PLSP2/B3A0P3 and CCD1/B3A0Q3 yielded more than three phosphopeptides with variable site-occupancy (Additional file 4: Table S4). Of these, Coiled-coil domain-containing protein 1 (CCD1)/B3A0Q3 was already shown to be extremely acidic previously [18], a feature that is enhanced by phosphorylation. This may be taken as a further indication of a very important, but as yet not understood, role of this protein in *Lottia* shell assembly.

Taking into account the number of phosphorylation sites and site occupancy, CCD1/B3A0Q3 may be considered as the major phosphoprotein of the *Lottia gigantea* shell matrix. We want to point out, however, that densely phosphorylated proteins with highly repetitive sequences, such as dentin phosphoryn, which contains almost exclusively aspartic acid, asparagine and phosphoserine [2], require special techniques to be identified and may be missing from our analysis.

A search for sequences including phospho sites for known kinase motifs indicated that approximately one third (16 of 46) of the unique S/T phospho sites comply with the Fam20C recognition site S-x-E or related motifs (S/T-x-E/D/pS/pT) [3,4]. This percentage is in good agreement with the approximately 24% of human secreted phosphoproteins modified at the serine of the canonical FAM20C motif S-x-E [6]. However, much less is known about phosphorylation in invertebrate secreted proteins and the kinases involved. Therefore it is unknown whether these recognition sites are conserved between vertebrates and invertebrates. Five of the sites identified are in agreement with the typical casein kinase 2 motif S-x-x-E also modified in the mammalian mineralization-inhibiting protein osteopontin, and ten sites comply with the casein kinase 1 motif (D/E)_n_-x-x-S/T [1] indicating that secreted or membrane-bound kinases with casein-kinase-like activity are involved. Evidence for such kinases is summarized in [5,6].

## Conclusions

**Figure 2:**
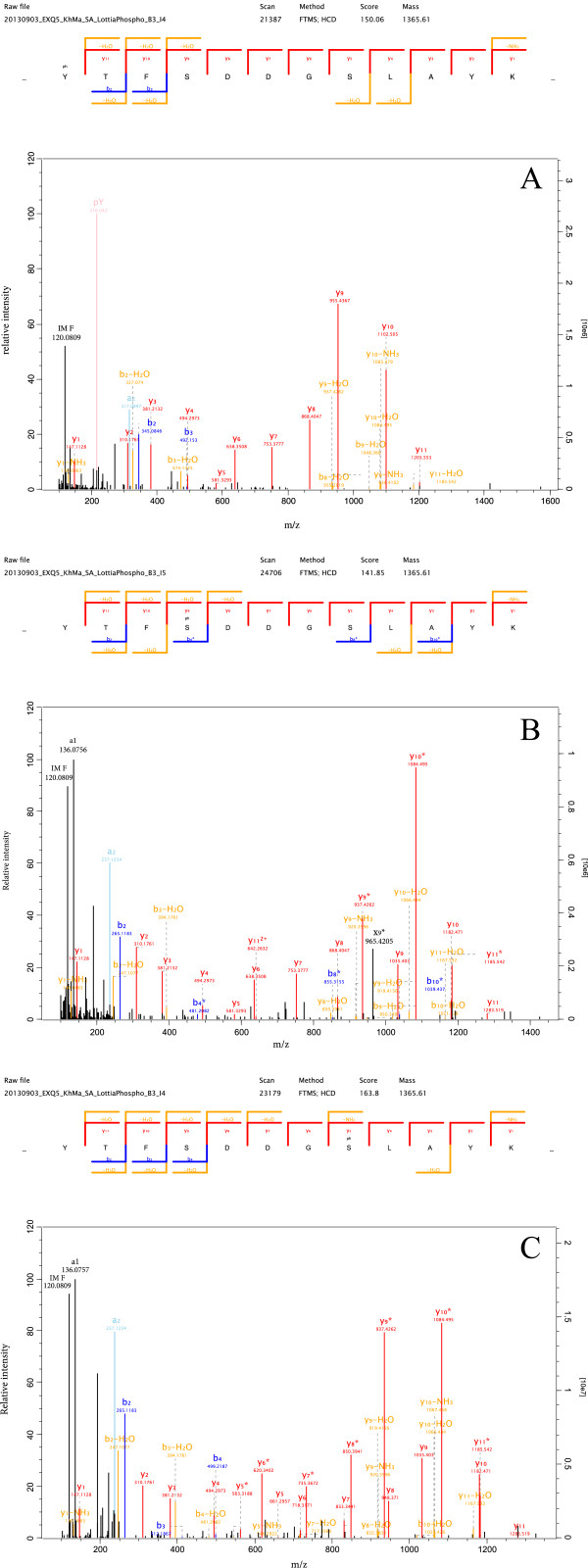
**An example of different partially occupied phospho sites in one sequence.** This peptide occurs in the sequence of DGLSP/B3A0P1/Lotgi1|162078 (Aspartate-, glycine-, lysine- and serine-rich protein, aa324-335). **A**, peptide variant with phosphotyrosine identified by an uninterrupted series of y-ions for the rest of the sequence and the very intense diagnostic pY immonium ion at m/z 216.042. Expert annotations [29] were omitted, except for the major peak at m/z 120.0809 (phenylalanine immonium ion), to keep the spectrum clear. The doubly charged peptide ion was measured with a mass error of −0.014 ppm. PEP and phosphphorylation site localization probability were calculated by MaxQuant to be 8.96e-93 and 0.999. **B**, this time S_4_ was determined as the phosphorylation site in an uninterrupted series of y-ions from y1 to y11. The mass error was −0.490 ppm, PEP was 1.16e-54 and the localization probability was 1.00. Major peaks at m/z 120.0809 and 136.0756 were annotated by the MaxQuant Expert system as the phenylalanine immonium ion and the a1-ion. A major peak at m/z 192.1016 was not annotated. Expert annotations of most of the minor peaks are omitted for clarity. **C**, a third phosphorylation site at S_8_ was detected with a localization probability of 1.00 in still another variant of this peptide measured with a mass error of 0.531 ppm and with a PEP of 3.28e-164. Again, most expert annotations are omitted. *, ions showing a loss of H_3_PO_4_ from phosphoserine. Y-ions are shown in red, b-ions are shown in blue, b-or y-ions with a loss of ammonia or water are in orange, the a_2_ ion is shown in light blue, black identifies ions without annotation unless the annotation is shown on top of the peak.

Our approach to proteomes of invertebrate biominerals consists of washing the biominerals with hypochlorite in a less stringent way than proposed recently [59] to preserve extra-crystalline matrix components, and to identify as many proteins as possible after in-gel digestion of slices of the entire gel [17] irrespective of staining intensity, or after in-solution digestion using filter-aided sample preparation (FASP) [20]. Included in protein identification is quantitation, which was done using exponentially modified protein abundance index (emPAI) [41] previously [17], but was recently superseded [60] in favor of the more accurate automated iBAQ method [19] as implemented in more recent versions of MaxQuant. We believe that this approach is well suited to identify candidates for functional matrix proteins, most likely found among the most abundant components, while retaining all of the information about trace components, irrespective of whether these may have a function in biomineralization or not, and irrespective of whether they are intra-crystalline or belong to the extra-crystalline matrix. Proteins predominantly located intracellularly, such as cytoskeletal components, ribosomal proteins, proteasome subunits or cytoplasmic enzymes, belong to the minor components of the *Lottia* shell proteome (Additional file 2: Table S2) constituting only an insignificant fraction of the total. However, the identification and quantitation of such proteins may also depend in some way on the biomineral examined, the instrumentation used, and the washing procedures applied to the shell and we agree with others [59,61] that the mere presence of such proteins in the matrix sample does certainly not imply a function.The group of major proteins also contains several phosphoproteins. Those yielding high-occupancy phospho sites and/or many phosphorylated sequence-unique peptides were already identified without prior phosphopeptide enrichment in a general survey. However, subtleties such as the occurrence of different sites with high localization probability within one peptide sequence (Figure 2) are more likely detected with the higher copy numbers usually provided by phosphopeptide-enriched samples. Nevertheless, inclusion of phosphorylation among the variable modifications in general studies of low complexity proteomes may give an overview of what to expect with phosphopeptide-enriched samples and may provide a rough estimate of phospho site occupancies.

## Abbreviations

Aa: Amino acid; emPAI: Exponentially modified protein abundance index; FDR: False discovery rate; HCD: Higher-energy collision-induced decomposition; iBAQ: Intensity-based absolute quantification; PEP: Posterior error probability; TFA: Trifluoroacetic acid.

## Competing interests

The authors declare that they have no competing interests.

## Authors’ contributions

KM conceived the study, performed sample preparation and data acquisition. EE collected and mechanically cleaned *Lottia* shells and helped with database search and annotation. All authors took part in the design of the study and were critically involved in manuscript drafting. Both authors read and approved the final manuscript.

## Supplementary Material

Additional file 1: Table S1 This table shows the complete list of identified proteins/protein groups including identifications that were not accepted following closer inspection, for instance because only one peptide was sequenced with insufficient sequence coverage. The table includes relevant parameters as, for instance, additional accession numbers for protein groups, scores or molecular weight of predicted proteins. Due to the simultaneous use of two databases and the high redundancy of the AllModels database some few groups contained so many similar entries that the Excel program created extra cells to accommodate all data. This disrupted the regular pattern of lines and columns of the sheet. However, the start of new groups is easily recognizable by >jgi|Lotgi1 followed by the accession code. Click here for file

Additional file 2: Table S2 In contrast to Table S1 this table only lists accepted protein/protein group identifications. Click here for file

Additional file 3: Table S3 This MaxQuant output table shows all peptides leading to identifications in Table S1, their sequences, scores, and other relevant parameters. Due to the simultaneous use of two databases and the high redundancy of the AllModels database some peptides appeared in so many similar entries that the Excel program created extra cells to accommodate all data. This disrupted the regular pattern of lines and columns of the sheet. However, the start of new peptide entries is clearly recognizable by the peptide sequence. Peptides appear in alphabetical order. Click here for file

Additional file 4: Table S4 List of identified and accepted phosphopeptides and phosphoproteins from the general proteomic survey and from analysis of phosphopeptide-enriched samples. Click here for file

Additional file 5: Table S5 This table essentially contains the MaxQuant Phospho(STY)Sites output file with all relevant parameters such as sequences, scores, and localization probabilities. Click here for file
