# Supplementary material for: The Lottia gigantea shell matrix proteome: re-analysis including MaxQuant iBAQ quantitation and phosphoproteome analysis
Source: Proteome Sci. 2014 May 18;12:28. doi: 10.1186/1477-5956-12-28 (PMC4094399; doi:10.1186/1477-5956-12-28)
Supplement: Additional file 2: Table S2 — In contrast to Table S1 this table only lists accepted protein/protein group identifications. [file 1477-5956-12-28-S2.docx]

**Table S2**

**Accepted *Lottia gigantea* shell matrix protein identifications (method B)**

| **Protein** | **Accession** | **emPAI**  **soluble ^1^** | **emPAI**  **insoluble ^1^** | **% of total**  **(iBAQ) ^2^** |  |
| --- | --- | --- | --- | --- | --- |
|  |  |  |  |  |  |
| Ubiquitin/Polyubiquitin | Lotgi1\|126004  Lotgi1\|66982 | 14.8 | 24.1 | 0.0043 |  |
| Uncharacterized protein/similar to hyaluronidase; domain: DUF297 | Lotgi1\|163670  Lotgi1\|101223 | 6.7 | 3.6 | 0.0022 |  |
| Similar to proline iminopeptidase | Lotgi1\|224583  Lotgi1\|101230 | - | 0.5 | <0.0001 |  |
| Similar to nucleoside diphosphate kinase B | Lotgi1\|205662  Lotgi1\|101477 | - | 2.2 | 0.0002 |  |
| Similar to histone H2A | Lotgi1\|181153  Lotgi1\|99863 | 3.6 | 1.2 | 0.0094 |  |
| Similar to ras-related Rab-1A/10 | Lotgi1\|205560  Lotgi1\|75443 | - | 0.4 | 0.0002 |  |
| Uncharacterized protein; domain: aerolysin_like_toxin | Lotgi1\|103152  Lotgi1\|75342 | nd | 1.3 | 0.0001 |  |
| Similar to chitobiase/chitinase | Lotgi1\|209107  Lotgi1\|64421 | 2.9 | 9.0 | 0.0050 |  |
| Similar to proteasome subunit β-type | Lotgi1\|181139  Lotgi1\|97074 | - | 0.8 | <0.0001 |  |
| Similar to glutathione peroxidase | Lotgi1\|97333  Lotgi1\|66260 | 0.9 | - | <0.0001 |  |
| Paramyosin | Lotgi1\|153233  Lotgi1\|71570 | 1.7^3^ | - | 0.0002 |  |
| Similar to α-N-acetylgalactosaminidase | Lotgi1\|209340  Lotgi1\|64864 | 0.5 | 3.6 | 0.0011 |  |
| Similar to gelsolin; shares peptide with Lotgi1\|74295 | Lotg1\|105757  Lotgi1\|73507 | - | 7.4 | 0.0024 |  |
| Similar to lipoma HMGIC fusion partner-like 3; (77% identity to K1Q2M6_CRAGI) | Lotgi1\|105456  Lotgi1\|105860 | - | 2.2^3^ | <0.0001 |  |
| Similar to profilin; N-term acetyl-Ser | Lotgi1\|183446  Lotgi1\|78156 | 2.2 | 3.6 | 0.0005 |  |
| Similar to arginine kinase | Lotgi1\|183052  Lotgi1\|55275 | 1.1 | 0.5 | <0.0001 |  |
| Uncharacterized protein; 10% Phe; pI:5.0 | Lotgi1\|154166  Lotgi1\|78201 | - | 3.1 | 0.0002 |  |
| Similar to rho GDP dissociation factor | Lotgi1\|183614  Lotgi1\|229322 | - | 20.5 | 0.0011 |  |
| Similar to ezrin/radixin/moesin | Lotgi1\|106937  Lotgi1\|52951 | 2.8 | 7.9 | 0.0019 |  |
| Similar to proteasome subunit α-type | Lotgi1\|229407  Lotgi1\|96234 | - | 1.9 | 0.0001 |  |
| Similar to fascin; domains: fascin | Lotgi1\|108036  Lotgi1\|92119 | - | 3.2 | 0.0001 |  |
| Similar to protein disulfide isomerase | Lotgi1\|184255  Lotgi1\|64044 | - | 1.9 | 0.0004 |  |
| Similar to ribosomal protein L14 | Lotgi1\|229862  Lotgi\|68702 | - | 1.2 | <0.0001 |  |
| Similar to cAMP-regulated protein-like; domain: cofilin/ADF; N-term: acetyl-Ser | Lotgi1\|203293  Lotgi1\|229823 | - | 2.7 | 0.0004 |  |
| Similar to Rab6 | Lotgi1\|230007 | - | 1.9 | 0.0002 |  |
| Similar to FAM20C/DMP4 | Lotgi1\|156599  Lotgi1\|92774 | 7.9 | 1.5 | 0.0064 |  |
| Similar to proteasome subunit α | Lotgi1\|212487  Lotgi1\|74910 | - | 0.5 | <0.0001 |  |
| Similar to HSP70 | Lotgi1\|177837  Lotgi1\|156848 | - | 0.3 | 0.0004 |  |
| Similar to glutaminyl-peptide cyclotransferase; domain: peptidase_M28 | Lotgi1\|206060  Lotgi1\|94461 | 0.5 | 2.9 | 0.0020 |  |
| Similar to phosphoglycerate kinase | Lotgi1\|212640  Lotgi1\|92565 | - | 0.6 | <0.0001 |  |
| Similar to ribosomal protein S2 | Lotgi1\|203487  Lotgi1\|112000 | - | 0.7 | 0.0004 |  |
| Similar to peptidyl-prolyl cis/trans isomerase B | Lotgi1\|212757  Lotgi1\|96960 | - | 3.3 | 0.0008 |  |
| Antistasin-like protein; domains: multiple antistasin | Lotgi1\|113221  Lotgi1\|73534 | - | 0.8 | 0.0007 |  |
| Uncharacterized protein; domains: β-ketoacyl synthase, acyl transferase, NAD(P)-binding, β-ketoacyl reductase | Lotgi1\|158379  Lotgi1\|113596 | - | 0.2 | 0.0001 |  |
| Similar to quinone reductase/ζ-crystallin | Lotgi1\|187129  Lotgi1\|65433 | - | 0.9 | 0.0001 |  |
| Similar to twisted gastrulation/Glucose-fructose oxidoreductase domain-containing protein 1 (K1PTI3_CRAGI); domain: Tsg83216 | Lotgi1\|231460  Lotgi1\|159250 | 1.5 | - | 0.0002 |  |
| Similar to hephaestin/ceruloplasmin; domain: multicopper oxidase, cupredoxin; previously Lotgi1\|83160 | Lotgi1\|83216 | 6.0 | 15.2 | 0.0347 |  |
| Uncharacterized protein; domain: SH3, PH | Lotgi1\|115450 | - | 0.8 | 0.0001 |  |
| Similar to purple acid phosphatase_like; domain: MPP_PAPs | Lotgi\|150028  Lotgi1\|93785 | - | 0.3 | <0.0001 |  |
| Similar to MEC2; domains: band_7, stomatin | Lotgi1\|214616  Lotgi1\|73436 | - | 0.5 | <0.0001 |  |
| Similar to cytosolic malate dehydrogenase | Lotgi1\|188534  Lotgi1\|66124  B8R5H1_LOTGI | - | 2.2 | 0.0006 |  |
| Similar to gelsolin | Lotgi1\|214936  Lotgi1\|74295 | - | 0.9 | 0.0001 |  |
| Similar to proteasome subunit α | Lotgi1\|232029  Lotgi1\|86051 | - | 1.2 | <0.0001 |  |
| Uncharacterized protein; domain: esterase_lipase superfamily, Ndr | Lotgi\|150144  Lotgi1\|72677 | - | 1.0 | 0.0011 |  |
| Similar to 6-phosphogluconate dehydrogenase | Lotg1i\|150160  Lotgi1\|189066 | - | 0.3 | <0.0001 |  |
| Similar to ADP,ATP carrier protein | Lotgi1\|232343  Lotgi1\|75082 | - | 0.7 | 0.0001 |  |
| Similar to signal sequence receptor β-like protein; domain: translocon-associated β | Lotgi\|118304  Lotgi1\|87003 | - | 2.2 | 0.0002 |  |
| Similar to 84kDa HSP/HSP90A | Lotgi1\|161608  Lotgi1\|83953 | - | 0.2 | 0.0002 |  |
| Similar to PLSP2_LOTGI (aa1-107 61% identity to aa356-462); shares peptides with  Lotgi\|162078 | Lotgi1\|99791  Lotgi1\|119827 | 30.6 | 176.8 | 0.0127 |  |
| Similar to arrestin/β-arrestin-1 | Lotgi\|120072  Lotgi1\|92353 | - | 1.6 | 0.0002 |  |
| Similar to S-formylglutathione hydrolase | Lotgi\|120210  Lotgi1\|74987 | - | 0.3 | <0.0001 |  |
| 78kDa glucose-regulated protein/HSP70 | Lotgi1\|216416  Lotgi1\|63182 | - | 0.3 | 0.0001 |  |
| Similar to thioredoxin peroxidase 2 | Lotgi1\|150310  Lotgi1\|76343 | - | 0.8 | 0.0003 |  |
| Similar to tropomyosin | Lotgi1\|233095  Lotgi1\|233094  Lotgi\|82394 | 1.0 | - | 0.0001 |  |
| Similar to ATP synthase subunit α | Lotgi1\|206617  Lotgi1\|93217 | - | 0.9 | 0.0002 |  |
| Similar to si:dkey-222f8.3/ endoribonuclease; domain: XendoU | Lotgi1\|216792  Lotgi1\|74510 | - | 1.4 | 0.0001 |  |
| Similar to ribosomal protein L12 | Lotgi1\|217320  Lotgi1\|163295 | 1.4 | 3.2 | 0.0004 |  |
| Similar to fructose-bisphosphate aldolase | Lotgi1\|217342  Lotgi1\|233385 | - | 1.7 | 0.0001 |  |
| Similar to ribosomal protein L9 | Lotgi1\|217766  Lotgi1\|97336 | - | 1.8 | 0.0001 |  |
| Similar to polypeptide N-acetylgalactosaminyl transferase; domains: ricin_B_lectin, pp_GalNAc-T | Lotgi\|123129  Lotgi1\|178843 | - | 0.2 | <0.0001 |  |
| Similar to D-glucuronyl C5-epimerase; domain: C5_epim_C | Lotgi\|123168  Lotgi1\|178848 | - | 0.8 | 0.0001 |  |
| Similar to glutathione-S-transferase µ | Lotgi1\|233779  Lotgi1\|74361 | - | 5.0 | 0.0001 |  |
| Similar to peroxiredoxin-2; domain: alkyl hydroxyperoxide reductase | Lotgi1\|123611  Lotgi1\|218000 | - | 0.2 | 0.0030 |  |
| Similar to prohibitin | Lotgi1\|204240  Lotgi1\|94763 | - | 0.4 | <0.0001 |  |
| Similar to peptidylprolyl isomerase/FKBP2 | Lotgi1\|192237  Lotgi1\|78513 | 4.2 | 0.9 | 0.0018 |  |
| Similar to Ser/Thr-protein phosphatase | Lotgi1\|200510  Lotgi1\|95568 | - | 0.7 | <0.0001 |  |
| Similar to gelsolin | Lotgi1\|192582  Lotgi1\|178963 | - | 4.0 | 0.0004 |  |
| Similar to actin-related 2/3 complex subunit 4/ARP23 complex 20 kDa subunit | Lotgi1\|204359  Lotgi1\|179062 | - | 1.0 | 0.0001 |  |
| Similar to rab GDP dissociation inhibitor/Rab GDI protein | Lotgi1\|218952  Lotgi1\|93457 | - | 2.2 | 0.0002 |  |
| Similar to annexin, type IV | Lotgi1\|219492  Lotgi1\|72948 | - | 1.8 | 0.0001 |  |
| Similar to defender against apoptotic cell death 1 | Lotgi1\|193969  Lotgi1\|89295 | - | 2.2 | <0.0001 |  |
| Uncharacterized protein/similar to frizzled; domain: CRD_FZ/frizzled Cys-rich | Lotgi1\|89037  Lotgi1\|234883 | 2.7 | - | 0.0005 |  |
| Similar to guanine nucleotide-binding protein G, α-subunit | Lotgi1\|194019  Lotgi1\|82703 | - | 1.5 | 0.0001 |  |
| Similar to transmembrane emp24 domain-containing protein 9 | Lotgi1\|128992  Lotgi1\|167121 | - | 1.4 | <0.0001 |  |
| Uncharacterized protein; domain: RHOD (rhodanese) superfamily; 16% Glu; pI 4.7 | Lotgi1\|129390  Lotgi1\|98795 | 5.8 | 2.2 | 0.0088 |  |
| Similar to glutathione-S-transferase | Lotgi1\|195517  Lotgi1\|76094 | - | 0.4 | <0.0001 |  |
| Similar to cathepsin L-like cysteine proteinase | Lotgi1\|221163  Lotgi1\|74117 | - | 0.3 | 0.0001 |  |
| Similar to dipeptidyl peptidase 1/cathepsin C | Lotgi1\|221343  Lotgi1\|221347 | - | 0.4 | 0.0003 |  |
| Similar to microsomal glutathione S-transferase; domain: membrane-associated proteins in eicosanoid and glutathione metabolism (MAPEG) | Lotgi1\|196152  Lotgi1\|78173 | - | 0.9 | 0.0001 |  |
| Similar to voltage-dependent anion channel 2-like protein/porin | Lotgi1\|168464  Lotgi1\|54458 | - | 1.6 | 0.0006 |  |
| Similar to thioredoxin-domain containing protein/ERp44; domain: thioredoxin-related | Lotgi1\|131935  Lotgi1\|93449 | 4.4 | 6.8 | 0.0031 |  |
| Similar to saccharopine dehydrogenase b134213 | Lotgi1\|222130  Lotgi1\|72095 | - | 0.6^3^ | <0.0001 |  |
| Similar to F-actin-capping protein subunit α | Lotgi1\|196809  Lotgi1\|55756 | - | 1.5 | 0.0001 |  |
| Similar to programmed death protein; domain: DUF1241 superfamily | Lotgi1\|168922  Lotgi1\|96940 | - | 0.9 | <0.0001 |  |
| Similar to DnaJ; domains: DnaJ N-term, TPR repeat | Lotgi1\|151060  Lotgi1\|53563 | 1.0 | - | 0.0002 |  |
| Similar to proteasome subunit β | Lotgi\|132853  Lotgi1\|56588 | - | 1.2 | <0.0001 |  |
| Similar to guanine nucleotide-binding protein subunit β; N-term acetyl-Ser | Lotgi1\|236462  Lotgi1\|82830 | - | 1.0 | 0.0001 |  |
| Similar to 14-3-3 ζ protein | Lotgi1\|222442  Lotgi1\|96882 | - | 2.4 | 0.0001 |  |
| Similar to translocon-associated protein subunit δ | Lotgi1\|204770  Lotgi1\|87465 | - | 2.2 | 0.0003 |  |
| Similar to dermatopontin/hemagglutinin/ amebocyte aggregation factor | Lotgi1\|133595  Lotgi1\|58521 | - | 6.7 | 0.0009 |  |
| Similar to sulfatase | Lotgi1\|197818  Lotgi1\|134213 | 0.4 | - | 0.0001 |  |
| Similar to Rab 7 | Lotgi1\|223383  Lotgi1\|134889 | - | 0.6 | 0.0001 |  |
| Similar to intermediate filament protein | Lotgi1\|204921  Lotgi1\|53511 | - | 0.3 | <0.0001 |  |
| Similar to growth differentiation factor 11/myostatin; domains: TGF-β | Lotgi1\|82990  Lotgi1\|179936 | 0.9 | - | <0.0001 |  |
| Similar to peptidyl-prolyl cis/trans isomerase; N-term: acetyl-Ala | Lotgi1\|223760  Lotgi1\|97345 | - | 1.8 | 0.0004 |  |
| Similar to Pi-class glutathione S-transferase | Lotgi1\|237303  Lotgi1\|85707 | - | 0.8^3^ | <0.0001 |  |
| Similar to ribosomal protein S3 | Lotgi1\|223917  Lotgi1\|87178 | - | 0.4 | <0.0001 |  |
| Similar to prohibitin | Lotgi1\|237446  Lotgi1\|96017 | - | 0.6 | <0.0001 |  |
| Similar to acidic ribosomal protein P0 | Lotgi1\|237709  Lotgi1\|95414 | - | 1.6 | 0.0003 | (P) |
| Uncharacterized protein/similar to peflin; domains:EFh | Lotgi1\|199248  Lotgi1\|56339 | nd | nd | <0.0001 |  |
| Similar to ribosomal protein L5 | Lotgi1\|224562  Lotgi1\|96972 | - | 0.5 | <0.0001 |  |
| Similar to DnaJ heat shock protein; domain: HSP DnaJ N-term, C-term | Lotgi1\|138864  Lotgi1\|54701 | 1.8 | - | 0.0019 |  |
| Similar to emp24 domain-containing protein | Lotgi1\|172331  Lotgi1\|59243 | - | 1.2 | <0.0001 |  |
| Similar to Rab11 | Lotgi1\|225073  Lotgi1\|96502 | - | 1.3 | <0.0001 |  |
| Similar to EGF receptor | Lotgi1\|238156  Lotgi1\|56548 | 0.4 | - | <0.0001 |  |
| Triosephosphate isomerase | Lotgi1\|238326  Lotgi1\|57182 | - | 6.0 | 0.0002 |  |
| Similar to malate dehydrogenase | Lotgi1\|225558  Lotgi1\|55305 | - | 0.8 | 0.0001 |  |
| Similar to protein Mo25 | Lotgi1\|238430  Lotgi1\|95282 | - | 0.8 | 0.0001 |  |
| Similar to peroxiredoxin 6; domain: AhpC_TSA/peroxiredoxin | Lotgi1\|225601  Lotg1\|140378 | nd | nd | <0.0001 |  |
| Similar to cathepsin D; Lotgi1\|176001 possibly a fragment of 173095 | Lotgi1\|173095  Lotgi1\|83423 | - | 0.9 | 0.0002 |  |
| Similar to β-catenin | Lotgi1\|205205  Lotgi1\|92025 | 0.1 | - | 0.0002 |  |
| Similar to FKBP-type peptidyl-prolyl cis/trans isomerase | Lotgi1\|238515  Lotgi1\|96742 | 5.3 | - | 0.0006 |  |
| Similar to aldose reductase (fragment) | Lotgi1\|140773 Lotgi1\|67044 | - | 0.8 | 0.0003 |  |
| Similar to angiopoietin-related protein 6; 55% identity to aa413-618 of K1PSU6_CRAGI; domain: fibrinogen_C | Lotgi1\|140992 | nd | nd | 0.0003 |  |
| Similar to short-chain dehydrogenase/reductase SDR | Lotgi1\|141698  Lotgi1\|63637 | - | 1.3 | 0.0002 |  |
| Similar to N-acylsphingosine amidohydrolase; domain: CBAH/acid ceramidase-like/Ntn_AC_NAAA | Lotgi1\|142206  Lotgi1\|180498 | - | 0.3 | 0.0001 |  |
| Similar to transaldolase | Lotgi1\|142681  Lotgi1\|93946 | - | 1.1 | 0.0001 |  |
| Similar to ATP synthase subunit β | Lotgi1\|201878  Lotgi1\|180584 | - | 3.9 | 0.0006 |  |
| Similar to small G protein/Ras-like protein | Lotgi1\|226838  Lotgi1\|67116 | - | 1.8^3^ | 0.0001 |  |
| Similar to elongation factor 1α | Lotgi1\|239271  Lotgi1\|55114 | - | 0.6 | 0.0005 |  |
| Similar to 14-3-3 protein B | Lotgi1\|205379  Lotgi1\|66022 | 0.6 | 3.0 | 0.0024 |  |
| Similar to carbonic anhydrase | Lotgi1\|205401  Lotgi1\|96190 | 0.8 | - | 0.0001 |  |
| Similar to carbonic anhydrase; domain: α-carbonic anhydrase | Lotgi1\|66515  Lotgi1\|180640 | 11.9 | - | 0.0047 |  |
| Similar to villin-1 fragments | Lotgi1\|144968  Lotgi1\|180698  Lotgi1\|227360  Lotgi1\|227362 | -  - | 1.7  2.3 | 0.0009 |  |
| Similar to fimbrin/plastin; domains: EFh, calponin, actinin | Lotgi1\|203020  Lotgi1\|228175 | - | 3.2 | 0.0011 |  |
| Similar to calumenin; domains: multiple EFh | Lotgi1\|103470  Lotgi1\|152613 | 1.7 | - | 0.0011 |  |
| Similar to Ran-1-prov | Lotgi\|104069  Lotgi1\|228472 | - | 0.6 | 0.0001 |  |
| Uncharacterized protein; domain: SOUL | Lotgi1\|229543 | 14.8 | 30.6 | 0.0825 |  |
| Uncharacterized protein; domains: peptidase C26/γ-glutamyl hydrolase | Lotgi1\|229608  Lotgi1\|155431 | - | 1.3 | 0.0001 |  |
| Uncharacterized protein; domain: IG_like (c2) | Lotgi1\|108898  Lotgi1\|229726 | 5.3 | 1.0 | 0.0058 |  |
| Similar to intermediate filament protein | Lotgi1\|109284  Lotgi1\|229925 | - | 1.1 | 0.0001 |  |
| Similar to osteonectin/BM-40/SPARC, domains: EFh, KAZAL | Lotgi1\|109908  Lotgi1\|230175  Lotgi1\|176394 | 16.8 | 9.0 | 0.0154 |  |
| Similar to synthenin; domains: PDZ | Lotgi1\|186095  Lotgi1\|230631 | - | 2.5 | 0.0002 |  |
| Uncharacterized protein; 11% Asn | Lotgi1\|203811  Lotgi1\|160216 | 1.3 | - | 0.0007 |  |
| Similar tochloride intracellular channel protein/glutathione-S-transferase C-term | Lotgi1\|189089  Lotgi1\|232272 | 1.3 | 7.5 | 0.0016 |  |
| Similar to thioredoxin; N-term: Ac-Ser | Lotgi1\|203919  Lotgi1\|232352 | - | 2.2 | 0.0004 |  |
| Similar to peptidyl-glycine α-amidating monooxygenase | Lotgi1\|204047  Lotgi1\|233030 | 4.4 | 0.8 | 0.0009 |  |
| Similar to short-chain dehydrogenase/reductase SDR | Lotgi1\|191692  Lotgi1\|163796 | - | 0.7 | <0.0001 |  |
| Similar to ferritin | Lotgi1\|178880  Lotgi1\|164282 | 16.8 | 16.8 | 0.0159 |  |
| Similar to multicopper oxidase/laccase-2; domain: multicopper oxidase type 1 | Lotgi1\|124263  Lotgi1\|164606 | 0.2 | 0.4 | 0.0003 |  |
| Similar to melanotransferrin; domains: transferrin | Lotgi1\|234865 | 0.3 | 1.9 | 0.0033 |  |
| Similar to translation initiation factor eIF4 | Lotgi1\|207101  Lotgi1\|179405 | - | 0.9 | <0.0001 |  |
| Similar to proactivator polapeptide (prosaposin) | Lotgi1\|221240  Lotgi1\|235753 | 0.2 | 0.7 | 0.0005 |  |
| Uncharacterized protein; domain: USP (universal stress protein)_like | Lotgi1\|204747  Lotgi1\|236343 | 0.9 | 25.8 | 0.0074 |  |
| GAPDH; shares 1 peptide with contaminant (bovine GAPDH) | Lotgi1\|222542  Lotgi1\|236520 | - | 3.6 | 0.0013 |  |
| β-tubulin | Lotgi1\|202077  Lotgi1\|226984 | 0.3 | - | 0.0028 |  |
| Uncharacterized protein/similar to calmodulin; domains: EFh | Lotgi1\|204915  Lotgi1\|237104 | 4.2 | 1.7 | 0.0004 |  |
| Na^+^/H^+^ exchange regulatory cofactor NHE-RF1; domains:PDZ | Lotgi1\|170528 | nd | nd | <0.0001 |  |
| Uncharacterized protein; domain: SOUL | Lotgi1\|205030  Lotgi1\|237594 | 41.2 | 16.8 | 0.0983 |  |
| Similar to cystatin; domains: cystatin | Lotgi1\|172187 | - | 1.8 | 0.0007 |  |
| Similar to epidymal secretory protein E1-like | Lotgi1\|226175  Lotgi1\|173629 | - | 4.2 | 0.0012 |  |
| Similar to alginate lyase | Lotgi1\|239189 | - | 24.1 | 0.0042 |  |
| Uncharacterized protein; 12% Pro 11% Leu, 10% Lys | Lotgi1\|227783 | 67.1 | 2.2 | 0.0462 |  |
| Uncharacterized protein; 27% Thr | Lotgi1\|227996 | 0.8 | 0.8 | 0.0002 |  |
| Similar to pleiotrophic membrane chitin-binding protein/chitin deacetylase; domain: polysaccharide deacetylase | Lotgi1\|181237  Lotgi1\|228005 | 1.1 | 1.3 | 0.0015 |  |
| Uncharacterized protein; domain: esterase/lipase/thioesterase | Lotgi1\|102397  Lotgi1\|228122 | - | 5.8 | 0.0044 |  |
| Uncharacterized protein; 20% Gly, 12% Leu | Lotgi1\|152688  Lotgi1\|152687 | 2.2 | - | 0.0028 |  |
| Uncharacterized protein; domain CCP (complement control protein) | Lotgi1\|152699 | 1.8 | - | 0.0011 |  |
| Similar to Pif97; 28% identical to PIF_PINFU: domains: vWA, chitin-binding, Con_A_ lectin | Lotgi1\|228264 | 2.5 | 71.0 | 0.1537 |  |
| Uncharacterized protein; 11% Pro | Lotgi1\|152799 | 718.7 | - | 0.0097 |  |
| Uncharacterized protein; signal peptide | Lotgi1\|152812 | nd | nd | <0.0001 |  |
| Uncharacterized protein; 18% Arg, 11% Ser; pI 11.7 | Lotgi1\|228385 | 9.0 | - | 0.0005 |  |
| Similar to thioester-containing protein/α2-macroglobulin | Lotgi1\|209261  Lotgi1\|209264 | 3.0 | 12.4 | 0.0172 |  |
| Uncharacterized protein; 10% Leu, 11% Ser | Lotgi1\|153653 | 0.4 | - | 0.0004 |  |
| Uncharacterized protein; domains: Cys-rich repeat (3x); 12% Gln, 13% Pro | Lotgi1\|228882 | 999.0 | 7.3 | 0.0835 |  |
| Similar to lustrin A fragment (45% identity to aa 6-201 of J7QAX0_PATVU); domains: WAP | Lotgi1\|228883 | nd | nd | 0.0002 |  |
| Uncharacterized protein; 11% Glu, pI 4.3; repeats in C-terminal half | Lotgi1\|154020 | 77.1 | 3.0 | 0.0974 | P* |
| Similar to protocadherin Fat4 (K1PTY5_CRAGI; 31-36% identity); domains: cadherin | Lotgi1\|106548  Lotgi1\|229125 | nd | nd | 0.0002 |  |
| Uncharacterized protein; domains: CLECT (25% identity to perlucin-like protein, PLCL_MYTGA), ZP_2 | Lotgi1\|229175 | 18.8 | 17.2 | 0.0313 |  |
| Uncharacterized protein | Lotgi1\|154423 | 1.7 | 9.0 | 0.0008 |  |
| Uncharacterized protein | Lotgi1\|154424 | 3.2 | 2.2 | 0.0009 |  |
| Uncharacterized protein; domains: cadherin repeats | Lotgi1\|229248  Lotgi1\|154545 | - | 0.2 | <0.0001 |  |
| Similar to meteorin-like protein (33% identity to K1P8K1_CRAGI); domain: meteorin_like/NTR | Lotgi1\|154590 | 1.1 | 9.0 | 0.0035 |  |
| Uncharacterized protein | Lotgi1\|154713 | 4.6 | 4.6 | 0.0035 |  |
| Uncharacterized protein; domains: FN3 | Lotgi1\|168990  Lotgi1\|229371 | 0.1 | - | <0.0001 |  |
| Uncharacterized protein; domain: carbohydrate –binding CBM_6/Galactose-binding | Lotgi1\|229427 | 0.9 | 0.5 | 0.0003 |  |
| Uncharacterized protein | Lotgi1\|229482 | 1.9 | 1.9 | 0.0009 |  |
| Uncharacterized protein; domain: PMP-22/EMP/MP20/claudin | Lotgi1\|229513 | - | 1.8 | 0.0001 |  |
| Similar to thioester-containing protein/CD109 antigen-like; domains: A2M_N, A2M_N_2 | Lotgi1\|229818 | 0.1 | 1.6 | 0.0010 |  |
| Similar to lipase; domains: esterase/lipase/thioesterase | Lotgi1\|109426  Lotgi1\|156157 | 0.7 | 1.2 | 0.0017 |  |
| Similar to sodium/potassium/calcium exchanger; domains: Na_Ca_ex; tm | Lotgi1\|98299  Lotgi1\|230017 | 0.8 | 6.5 | 0.0026 |  |
| Similar to pancreatic lipase-related; domain: lipase, tm; aa28-463 36% identity to aa327-755 of dentin sialoprotein (D7PVG8_ HUMAN) | Lotgi1\|98300  Lotgi1\|156200 | 1.5 | 9.0 | 0.0031 |  |
| Uncharacterized protein; 16% Q, 11% L, 13% K | Lotgi1\|156257 | nd | nd | 0.0182 |  |
| Uncharacterized protein; 15% Lys, 12% Leu; 3 ~75aa repeats | Lotgi1\|230068 | - | 10.5 | 0.0026 |  |
| 31% identity to aa17-381 of protocadherin Fat 4 (K1PTY5_CRAGI); domains: cadherin | Lotgi1\|176992  Lotgi1\|230170 | - | 3.6 | 0.0034 |  |
| Uncharacterized protein; domains: CLECT, CUB, sushi/SCR/CCP, EFh) | Lotgi1\|156525 | 0.3 | 0.2 | 0.0001 |  |
| aa766-2406 30% identity to signal peptide, CUB and EGF-like domain-containing protein 3 (K18890_CRAGI); domains: EGF, hyaline, tyrosine-protein kinase ephrin_like | Lotgi1\|230225 | nd | nd | <0.0001 |  |
| Uncharacterized protein; 12% Leu, 12% Lys, pI 9.4 | Lotgi1\|156601 | 1.2 | 1.2 | 0.0021 |  |
| Uncharacterized protein ; domain:WxxW | Lotgi1\|110884  Lotgi1\|230322 | 14.8 | 14.8 | 0.0032 |  |
| EFCB1/B3A0Q5 | Lotgi1\|230492  EFCB1_LOTGI | 516.9 | 6.2 | 0.0484 |  |
| Similar to calcium-binding protein; domain: EFh; 43% identity to EFCB1_LOTGI | Lotgi1\|230493 | 5.8 | 0.5 | <0.0001 |  |
| Proline-rich protein 2/B3A0R8 | Lotgi1\|230510  PRP2_LOTGI | 773.3 | 3.6 | 1.6679 |  |
| Uncharacterized protein; domains: Kazal | Lotgi1\|157133 | 1.7 | - | 0.0002 |  |
| Uncharacterized protein; signal seq | Lotgi1\|157305 | nd | nd | 0.0072 |  |
| Uncharacterized protein | Lotgi1\|230689 | 0.5 | - | 0.0001 |  |
| Uncharacterized protein, 13% Val | Lotgi1\|157680 | - | 0.9 | 0.0003 |  |
| Uncharacterized protein; 43% identity to EFCB2_LOTGI | Lotgi1\|157683 | nd | nd | 0.0392 |  |
| Uncharacterized protein, 38% identity to EFCB2_LOTGI | Lotgi1\|230731 | nd | nd | 0.0015 |  |
| Uncharacterized protein; 43% identity to EFCB2_LOTGI | Lotgi1\|157689 | 99.0 | - | 0.0455 |  |
| Uncharacterized protein; 49% identity to EFCB2_LOTGI | Lotgi1\|157690 | 9.0 | 0.3 | 0.0185 |  |
| Uncharacterized protein; 44% identity to EFCB2_LOTGI | Lotgi1\|230732 | 9.0 | - | 0.0019 |  |
| Uncharacterized protein; 11% Val | Lotgi1\|157827 | 9.0 | 20.5 | 0.0219 |  |
| Similar to pacifastin; domains: VWC/pacifastin | Lotgi1\|230854  Lotgi1\|176463  Lotgi1\|99757 | 45.4 | 5.8 | 0.2323 |  |
| Uncharacterized protein; 11% Pro, Gln-rich C-terminal motif | Lotgi1\|158113 | 5010.9 | 14.8 | 1.1870 |  |
| Uncharacterized protein | Lotgi1\|230881 | 13.7 | - | 0.0036 |  |
| Uncharacterized protein; 11% Ser, 10% Gln | Lotgi1\|158316 | 243.2 | 1.2 | 0.1011 |  |
| Uncharacterized protein; 12% Leu, 10% Ala | Lotgi1\|231009 | 9999.0 | 38.8 | 0.8712 |  |
| Uncharacterized protein; 11% Gly, 10% Ser | Lotgi1\|231010 | 12.6 | - | 0.0061 |  |
| Uncharacterized shell protein 16/B3A0R5 | Lotgi1\|231046  USP16_LOTGI | 358.4 | 15.7 | 2.0115 |  |
| Uncharacterized protein; 19% Gly, 12% Pro | Lotgi1\|231186 | 175.8 | - | 0.0311 |  |
| Similar to blastula protease 10 (aa261-575 35% identity to aa27-353); domain: peptidase M12A/astacin | Lotgi1\|89537  Lotgi1\|231231 | nd | nd | 0.0150 |  |
| Glycine-, glutamate- and proline-rich protein/B3A0P5 | Lotgi1\|114561  Lotgi1\|231311  Lotgi1\|213813  GEPRP_LOTGI | 9999.0 | 99.0 | 1.4483 | P* |
| Similar to chitin-binding peritrophin-A; domains: chitin-binding, Con_A_like | Lotgi1\|231395 | 6.7 | 5.0 | 0.0061 |  |
| Uncharacterized protein; aa42-160 90% identity to EFCB2 | Lotgi1\|231426 | 9.0 | 1.2 | 0.0059 |  |
| Uncharacterized protein/B3A0R9 | Lotgi1\|231427  EFCB2_LOTGI | - | 2.2 | 0.0001 |  |
| Uncharacterized protein/similar to conodipine-M α-chain; domain: partial Phospholip_A2 | Lotgi1\|159173  Lotgi1\|176428 | 2.4 | 0.7 | 0.0439 |  |
| Uncharacterized protein | Lotgi1\|159264 | 1.2 | 10.7 | 0.0239 |  |
| Similar to buccalin; sequence consists mainly of nineteen 14aa repeats KRGxDxf/yFxGQLG | Lotgi1\|159314 | 1.0 | - | 0.0005 |  |
| Uncharacterized protein; domains: ARM repeats, LDLRA_1 | Lotgi1\|115147  Lotgi1\|159329 | 14.2 | 4.3 | 0.0084 |  |
| Uncharacterized protein; 16% Pro, 10% Leu, 10% Ser | Lotgi1\|231509  Lotgi1\|159330 | 9.0 | - | 0.0016 |  |
| Uncharacterized protein; 26% Gln, 13% Leu, 12% Thr, pI 4 | Lotgi1\|159331 | 9.0 | - | 0.2175 |  |
| Uncharacterized protein; domains: FAD-linked oxidase, arabinono-1,4-lactone oxidase | Lotgi1\|159383 | - | 1.3 | 0.0007 |  |
| Uncharacterized protein; domains: SRCR, chitin-binding | Lotgi1\|159694 | 0.2 | - | <0.0001 |  |
| Uncharacterized protein | Lotgi1\|159735 | - | 1.3 | <0.0001 |  |
| Uncharacterized protein similar to ceruloplasmin; domains: multiple cupredoxin, multicopper oxidase, type 2 | Lotgi1\|115607  Lotgi1\|115527  Lotgi1\|159783  Lotgi1\|179818 | 10.8 | 67.1 | 0.0506 |  |
| Uncharacterized protein/similar to Leishmania proteophosphoglycan; domain: SEA; 27% Ser, 14% Thr | Lotgi1\|231862 | - | 0.7 | 0.0002 |  |
| Similar to YIPF6 (69% identity to K1QNR7_CRAGI) | Lotgi1\|230890 Lotgi1\|231865  Lotgi1\|160165 | - | 4.2 | 0.0006 |  |
| Uncharacterized protein; domains: multiple chitin-binding/peritrophin A | Lotgi1\|231869 | 29.9 | 3.7 | 0.0193 |  |
| Uncharacterized protein; domain: Chitin-binding_2/CBM_14 | Lotgi1\|160173 | 277.3 | 11.9 | 0.0359 |  |
| Uncharacterized protein; 13% Gln, 11% Gly, 11% Thr | Lotgi1\|160356 | 4.0 | 6.9 | 0.0271 |  |
| Similar to peptidase S8, S53, subtilisin, kexin, sedolisin | Lotgi1\|160410 | - | 1.2 | 0.0002 |  |
| Similar to PIF; 24% identity to PIF_PINFU; domains: vWA, chitin-binding | Lotgi1\|232022 | - | 0.5 | 0.0002 |  |
| Similar to acetylcholine-binding protein (30% identity to B3SNJ8_HALDH); domain: neurotransmitter-gated ion-channel | Lotgi1\|160701 | nd | nd | 0.0002 |  |
| Uncharacterized protein; 11% Ser | Lotgi1\|232714 | 1.9 | 1.6 | 0.0070 | P |
| Aspartate-, glycine-, lysine- and serine-rich protein/B3A0P1 (100% identity in matching regions)/peroxidase-1 | Lotgi1\|162078  Lotgi1\|75899  DGLSP_LOTGI | nd | nd | 16.7056 | P* |
| Uncharacterized protein; 100% identity in overlap with PLSP2/B3A0P3 | Lotgi1\|77105  Lotgi1\|232817  Lotgi1\|99852  PLPS2_LOTGI | 397.1 | 630.0 | 6.7971 | P* |
| PLSP3/peroxidase-3/B3A0Q8 | Lotgi1\|99809  Lotgi1\|232818  PLSP3_LOTGI | 214.4 | 463.2 | 0.2586 |  |
| Uncharacterized protein; domains: SEA, chitin-binding peritrophin A | Lotgi1\|232880 | - | 1.4 | 0.0007 |  |
| Uncharacterized protein; 12% Pro; (G/R)PP repeats at the C-terminus | Lotgi1\|162562 | 2.2 | 5.6 | 0.0025 |  |
| SCP domain-containing protein 1/B3A0P7 | Lotgi1\|233199  SCP1_LOTGI | 999.0 | 364.2 | 0.5279 |  |
| SCP domain-containing protein 2/B3A0P8 | Lotgi1\|233200  SCP2_LOTGI | >10,000 | 507.0 | 0.9684 |  |
| Uncharacterized protein; domains: CAP/allergen V5 | Lotgi1\|233201 | 1.2 | 1.0 | 0.0002 |  |
| Uncharacterized protein; 14% Ser, 10% Thr | Lotgi1\|162861 | 7.5 | 1.2 | 0.0079 |  |
| Uncharacterized protein; domains: α2-macroglobulin | Lotgi1\|162872  Lotgi1\|233231 | 6.8 | 12.7 | 0.0278 |  |
| Uncharacterized protein; domains: mucin2_WxxW | Lotgi1\|233299 | nd | nd | 0.0003 |  |
| Uncharacterized protein | Lotgi1\|233348 | 2.7 | 1.7 | 0.0012 |  |
| Similar to Nucb2b; 17% Glu, 11% Lys; domain: EFh | Lotgi1\|121860  Lotgi1\|217304 | 47.7 | - | 0.0064 |  |
| Uncharacterized protein; Pro/Ala- and His-rich motifs in C-term | Lotgi1\|233397  Lotgi1\|163339 | 516.9 | 12.9 | 0.2407 |  |
| Similar to cofilin/actin-depolymerizing factor | Lotgi1\|233408 | 1.3 | 3.3 | 0.0014 | (P) |
| Uncharacterized protein; 17% Ala | Lotgi1\|233451 | 3.6 | 3.6 | 0.0033 |  |
| Uncharacterized protein; 14% Gly, 13% Asn, 11%Arg, 11% Asp; the same? | Lotgi1\|233461  Lotgi1\|231310 | 9.9 | 2.9 | 0.0736 |  |
| Uncharacterized protein | Lotgi1\|163448 | 49.1 | 3.9 | 0.0496 |  |
| Similar to matrix metalloproteases-21; domain: ZnMc_MMP, hemopexin | Lotgi1\|122269  Lotgi1\|233533 | 0.4 | 2.3 | 0.0007 |  |
| Similar to guanine nucleotide-binding protein G(s) subunit alpha | Lotgi1\|163596  Lotgi1\|178803 | nd | 0.8^3^ | <0.0001 |  |
| Uncharacterized protein; domain: EFh, LEA; LUSP-10 | Lotgi1\|163637 | 18.3 | 99.0 | 0.2516 |  |
| Similar to ependymin-related protein 1/X-box-binding protein; domains: ependymin | Lotgi1\|233583 | - | 1.8 | 0.0013 |  |
| Similar to ribosomal protein S27 | Lotgi1\|191640  Lotgi1\|233598 | - | 3.6 | 0.0002 |  |
| 49% identity to aa89-307 of K1QHA4_CRAGI (uncharacterized), 48% identity to aa3-215 of Kyphoscoliosis peptidase (K1RL06_CRAGI) | Lotgi1\|233820 | 0.8^3^ | 0.5^3^ | <0.0001 |  |
| 32% identity to aa386-449 of Kunitz-type protease inhibitor 1 (K1S707_CRAGI); domains: BPTI_KUNITZ, signal seq | Lotgi1\|123902  Lotgi1\|233918 | 3.6^3^ | nd | 0.0001 |  |
| Similar to Kunitz-type protease inhibitor; domains: BPTI_KUNITZ | Lotgi1\|60578  Lotgi1\|218166 | nd | nd | 0.0003 |  |
| Uncharacterized protein; domains: GPS, GPCR/secretin_like, CLECT, tm | Lotgi1\|218330  Lotgi1\|234010 | nd | nd | 0.0004 |  |
| Uncharacterized protein; domains: Sushi/SCR/CCP | Lotgi1\|164755 | - | 1.7 | <0.0001 |  |
| Uncharacterized protein; 16% Ser, 11% Thr, 11% Pro | Lotgi1\|164956 | 0.9 | - | 0.0040 |  |
| Similar to Deleted in malignant brain tumors 1 protein (K1QA70_CRAGI); domains: MAM, SRCR, IG, signal seq | Lotgi1\|77902  Lotgi1\|69892  Lotgi1\|234272 | 1.4 | 2.2 | 0.0036 |  |
| Uncharacterized protein; domain: CD225 (interferon-induced transmembrane protein), Gln-rich N-term (~aa20-50, P[Q]_2-3_GY) | Lotgi1\|234302 | - | 30.6 | 0.0009 |  |
| Uncharacterized protein; 13% Ala, 11% Gly | Lotgi1\|234386 | 5.0 | 3.6 | 0.0260 |  |
| Uncharacterized protein | Lotgi1\|234387 | 141.5 | 69.2 | 0.3789 |  |
| Uncharacterized protein; domains: chitin-binding, (peritrophin A) | Lotgi1\|234405 | 23.2 | 3.1 | 0.0061 |  |
| Similar to actin; shares peptides with contaminant (bovine actin) | Lotgi1\|193218  Lotgi1\|219078  Lotg1\|239714  Lotgi1\|202971  Lotgi1\|215510 | 1.0 | 0.5 | 0.0391 |  |
| Uncharacterized protein/similar to *Haliotis* Latent transforming growth factor beta binding protein; 13% Cys, 10% Asn, 10% Gly | Lotgi1\|234488 | 12.3 | 4.6 | 0.0131 |  |
| Uncharacterized protein; 6 x 25aa repeats, signal peptide | Lotgi1\|234489 | nd | nd | 0.0022 |  |
| Similar to stanniocalcin (40% identity to B3TK27/E4W3F3_HALDV) | Lotgi1\|234508 | nd | 6.5^3^ | <0.0001 |  |
| Uncharacterized protein; domain: selenoprotein P | Lotgi1\|234596 | 1.7 | - | 0.0004 |  |
| Uncharacterized protein; 18% Asp, 16% Arg, 14% Gly; many GDDR and related repeats; domain: superoxide dismutase | Lotgi1\|234845 | 1.2 | - | 0.0003 |  |
| Similar to superoxide dismutase; domain: Cu-Zn superoxide dismutase , previously Lotgi1\|101611 | Lotgi1\|166131  Lotgi1\|127077  Lotgi1\|127098 | (38.8) | (38.8) | 1.0899 | P* |
| Similar to tyrosinase ^1^; 11% Pro; domain: tyrosinase; aa393-462 nine GPPVNP-type repeats | Lotgi1\|166196 | - | 0.5 | 0.0001 |  |
| Uncharacterized protein; domain: Sushi/SCR/CCP; 19% Gln, 11% Pro | Lotgi1\|234884  Lotgi1\|166202 | 189.5 | 5.3 | 0.1410 |  |
| aa151-448 96% identity to coiled-coil domain-containing protein 2/B3A0Q7 | Lotgi1\|234936  CCD2_LOTGI | nd | nd | 0.6703 |  |
| Uncharacterized protein; 10% Ser | Lotgi1\|166451 | 6.9 | 2.2 | 0.0067 |  |
| Proline-rich protein 1/B3A0Q1 | Lotgi1\|235497  PRP1_LOTGI | >10,000 | 463.2 | 12.2794 | P |
| EGF-like domain_containing protein 2/B3A0S3 | Lotgi1\|167423  ELDP2_LOTGI | nd | nd | 0.1874 |  |
| Similar to mannose receptor; domains: EGF, multiple C-type lectin, ZP_2 | Lotgi1\|235549  Lotgi1\|167426 | 0.8 | 1.0 | 0.0120 |  |
| Similar to interferon-γ-inducible lysosomal thiol reductase; domains: saposin A, GILT | Lotgi1\|235566 | 1.5 | - | 0.0003 |  |
| Threonine-rich protein TRP/B3A0R4 | Lotgi1\|235609  TRP_LOTGI | 4.4 | 3.0 | 0.0288 |  |
| Uncharacterized protein/LUSP-18; 15% Pro, 15% Thr | Lotgi1\|235610 | 3161.3 | 22.7 | 0.2038 |  |
| Glycine and tyrosine-rich protein/B3A0Q2 | Lotgi1\|235621  GTRP_LOTGI | 5178.5 | 25.8 | 0.7055 |  |
| Uncharacterized protein; some similarity (26% identity) to aa988-1887 of Trithorax group protein osa (K1PML0_CRAGI) | Lotgi1\|167578 | nd | nd | 0.0615 |  |
| Uncharacterized protein/similar to UDP-N-acetyl-a-D-galactosamine:polypeptide N-acetylgalactosaminyl-transferase | Lotgi1\|235690 | - | 1.3 | 0.0001 |  |
| Similar to FGF-binding protein; domain: FGFBP_1 | Lotgi1\|235694 | 5.0 | - | 0.0087 |  |
| Uncharacterized protein; domains: EFh | Lotgi1\|235797 | 2.0**^3^** | 1.5**^3^** | 0.0024 |  |
| Uncharacterized protein; 10% Asn, 18% Gln, 24% Pro; domains: Pro_rich_extensin | Lotgi1\|235812 | 13.3 | - | 0.0028 |  |
| Uncharacterized protein; 11% Ser | Lotgi1\|235865 | - | 1.8 | 0.0006 |  |
| Uncharacterized protein; 15% Ala | Lotgi1\|235969 | - | 4.6 | 0.0011 |  |
| Uncharacterized protein; domain: Hedgehog/DD-peptidase | Lotgi1\|235988 | 1.9 | 3.0 | 0.0042 |  |
| Uncharacterized shell protein 4/B3A0P9 | Lotgi1\|236183  USP4_LOTGI | 5178.4 | 137.9 | 0.7665 |  |
| Uncharacterized protein with 24% identity to aa748-3397 of K1RU24_CRAGI (notch); domains: EGF, thyroglobulin type-1, BPTI/Kunitz, Follistatin_like N-term, FN3, WAP | Lotgi1\|132911  Lotgi1\|236424 | 2.2 | - | 0.0008 |  |
| Uncharacterized protein; domains: SPOT2_related, signal seq | Lotgi1\|236449 | nd | nd | 0.0035 |  |
| Uncharacterized protein; 20% Pro, 10% Ala, 10% Arg, 10% Val | Lotgi1\|236689 | 21.8 | 5.1 | 0.0270 |  |
| Uncharacterized protein USP22/B3A0S0; aa1-195 99.5% identity to USP22 | Lotgi1\|236690  USP22_LOTGI | 13.7 | 2.2 | 0.0959 |  |
| Uncharacterized protein; 22% Pro, 13% Gln, 10% Ala | Lotgi1\|236691 | 30.6 | - | 0.0943 |  |
| Uncharacterized protein; domain: AdoMet_MTase superfamily | Lotgi1\|169491 | 0.5 | 0.5 | 0.0001 |  |
| Uncharacterized protein; 15% Pro | Lotgi1\|169557 | nd | nd | 0.0056 |  |
| Uncharacterized protein; 12% Pro | Lotgi1\|236812 | 1.3 | - | 0.0007 |  |
| Similar to peptidyl-prolyl cis/trans isomerase/B3A0R0 | Lotgi1\|222979  Lotgi1\|169679  PPI_LOTGI | 25.8 | 12.9 | 0.2419 |  |
| Uncharacterized protein; domains: EGF, Vitellinogen, FN | Lotgi1\|236952 | 0.1 | 0.6 | 0.0002 |  |
| Uncharacterized protein/similar to mannanase; domain: Glycohydrolase_26 | Lotgi1\|236955 | 0.5 | 0.8 | 0.0006 |  |
| Uncharacterized protein | Lotgi1\|169925 | 2.2 | - | 0.0001 |  |
| Uncharacterized/similar to K1QR68/K1QIP4/K1P9M8_CRAGI | Lotgi1\|237070 | nd | nd | <0.0001 |  |
| Uncharacterized protein; domain: LamG/concanavalin A-like lectin | Lotgi1\|237103 | - | 1.8 | 0.0005 |  |
| Similar to gastric intrinsic factor/ transcobalamin | Lotgi1\|237143 | 9.0 | 24.1 | 0.0108 |  |
| Uncharacterized protein; domain: reeler; 21% Thr, 15% Ser, 11% Pro | Lotgi1\|237152 | 0.5 | - | 0.0002 |  |
| Similar to tubulin α1/α2 | Lotgi1\|237161  Lotgi1\|223558  Lotgi1\|223561 | 0.7 | 2.4 | 0.0001 |  |
| Similar to tyrosine 3-monooxygenase activation protein/14-3-3 protein ε | Lotgi1\|207549  Lotgi1\|237480 | - | 4.4 | 0.0008 |  |
| Uncharacterized protein; 13% Ser, Glu-rich C-term motif; pI 4.4 | Lotgi1\|171084 | 99.0 | 3.6 | 0.1082 | P* |
| Similar to PIF; 41% identity to PIF_PINFU aa427-526 | Lotgi1\|237510  Lotgi1\|171086 | 176.8 | 41.2 | 0.1564 |  |
| Similar to thrombospondin type 1-containing protein; domains:TSP_1, ADAM-TS spacer, PLAC | Lotgi1\|237754 | 0.7 | - | 0.0001 |  |
| Uncharacterized protein | Lotgi1\|237778 | - | 2.2 | 0.0003 |  |
| Uncharacterized protein | Lotgi1\|171839 | 25.1 | - | 0.0158 |  |
| Proline-rich protein 3/B3A0S4 | Lotgi1\|237996  Lotgi1\|172116  PRP3_LOTGI | 99.0 | 99.0 | 0.3424 |  |
| Similar to nacrein-like protein/B3A0P2 | Lotgi1\|238082  CAH1_LOTGI | >10,000 | 8375.8 | 0.9603 |  |
| Uncharacterized protein; domain: DUF3421 | Lotgi1\|238094  Lotgi1\|172287 | 24.1 | 19.0 | 0.0251 |  |
| Uncharacterized protein; 23% Asn, 15% Pro, 15% Thr, 11% Ser | Lotgi1\|84059  Lotgi1\|172307 | 9.0 | - | 0.0031 |  |
| Uncharacterized protein; 11% Gln | Lotgi1\|238202 | nd | nd | 0.0043 |  |
| Uncharacterized protein; 10% Pro, 14% Thr | Lotgi\|172500 | nd | nd | 0.0054 |  |
| Uncharacterized protein; 23% Glu, 13% Asn, 13% Ser; multiple NQQF repeats | Lotgi1\|172698 | 8.0 | 0.6 | 0.0103 |  |
| Uncharacterized protein; aa1-138 100% identity to ASRP/B3A0S2 | Lotgi1\|238358  ASRP_LOGI | 41.2 | 55.2 | 0.1351 | P* |
| Similar to endochitinase (34% identity to aa3-300 of K1R034_CRAGI); domains: vWA_ECM, CBM_14 (peritrophin A) | Lotgi1\|238400 | 1.6 | 55.2 | 0.0252 |  |
| Uncharacterized protein | Lotgi1\|238415 | 0.6 | 0.3 | 0.0001 |  |
| Uncharacterized protein; domain: CBM_14 (chitin-binding)/peritrophin A; 27% identity to PIF_PINFU | Lotgi1\|173138 | 24.1 | 2510.9 | 0.8657 |  |
| Similar to collagen α4 (VI)/BMSP (49/46 % identity to aa280-620 of K1PTA3_CRAGI and G1UCX0_MYTGA); domains: VWA, 10% Ile, 10% Thr | Lotgi1\|140660  Lotgi1\|173139 | 41.2 | 2370.4 | 2.8102 |  |
| Similar to aminopeptidase N; domain: peptidase M1_APN_2 | Lotgi1\|238560 | 0.5 | 7.4 | 0.0448 |  |
| Methionine-rich protein /B3A0R7 | Lotgi1\|173200  MRP_LOTGI | 2237.7 | 34.5 | 1.4284 |  |
| Uncharacterized shell protein 5/B3A0Q0 | Lotgi1\|238831  USP5_LOTGI | 999.0 | 34.1 | 5.1093 |  |
| Similar to chloride-channel Ca-activated-2-like; domains: vWA, chloride channel, DUF1973 | Lotgi1\|238844 | - | 0.8 | <0.0001 |  |
| Uncharacterized protein; 15% Pro | Lotgi1\|174003 | 240.2 | 70.3 | 0.2189 |  |
| Similar to perlustrin/B3A0Q9; 43% identity to PLS_HALLA; domain: IGFBP_N_2 | Lotgi1\|238970  PLSLP_LOTGI | 99.0 | 9.0 | 0.1069 |  |
| Uncharacterized protein; 11% Cys, 10% Ser; similar to *Haliotis* perlustrin and PLSLP_LOTGI (34% identity) | Lotgi1\|174065 | <10,000 | 6308.6 | 0.8119 |  |
| Uncharacterized proteins; 14% Ser, 10% Gly; 93% identical | Lotgi1\|239005  Lotgi1\|239006 | 0.9 | 25.8 | 0.0183 |  |
| Uncharacterized protein; 22% Gln, 12% Pro | Lotgi1\|174203 | 5.8 | - | 0.0009 |  |
| Uncharacterized protein; 25% Gln, 17% Pro, 12% Val, 10% Asn, 10% Leu; short repeats (QQPXXVELNK) | Lotgi1\|142790  Lotgi1\|142814  Lotgi1\|174204 | 59.8 | 0.3 | 0.0052 |  |
| Uncharacterized protein; 11% Gln, 12% Pro | Lotgi1\|174205 | nd | nd | 0.0136 | P |
| Uncharacterized protein; some similarity to perlwapins; domains: WAP; 11% Asp, 13% Cys | Lotgi1\|143247  Lotgi1\|174418 | 3.6 | - | 0.0295 |  |
| Uncharacterized protein; domains: antistasin, WAP, 15% Cys, 11% Pro | Lotgi1\|201804  Lotgi1\|174421 | 0.9 | - | 0.0004 |  |
| Uncharacterized protein/LUSP-14 (Lotgi1\|174428); domain: chitin_binding_3 | Lotgi1\|226726  Lotgi1\|239129 | 11.1 | 1.2 | 0.1577 | P* |
| Uncharacterized protein; 10% Lys | Lotgi1\|239159 | 1.4 | 55.2 | 0.0086 |  |
| Glycine-rich protein/B3A0R2 | Lotgi1\|239170  GRP_LOTGI | >10,000 | 561.3 | 5.9134 |  |
| Uncharacterized protein; 18% Leu, 16% Gly, 12% Ala | Lotgi1\|239171 | 3.0 | - | 0.0003 |  |
| Uncharacterized protein; 18% Gly, 19% Met, 11% Leu; aa17-126 50% identity to aa27-128 of GMP_LOTGI | Lotgi1\|239173  Lotgi1\|174537 | 1.2**^3^** | - | 0.0213 |  |
| Glycine- and methionine-rich protein/ B3A0R1 | Lotgi1\|239174  GMP_LOTGI | >10,000 | 71.0 | 9.1395 |  |
| aa190-632 100% identity to CAH2/B3A0Q6 | Lotgi1\|239188  CAH2_LOTGI | 63.9 | 47.7 | 0.8795 |  |
| Glycine- and Serine rich protein-1/B3A0P6; fragment | Lotgi1\|239214  GSP1_LOTGI | nd | nd | 6.8154 | P |
| Uncharacterized protein; 12% Pro, 10% Ser | Lotgi1\|174652 | 13.2 | 0.4 | 0.0133 | (P) |
| Uncharacterized protein; 11% Pro, 11% Thr, 10% Val | Lotgi1\|239234 | nd | 0.7^3^ | <0.0001 |  |
| Uncharacterized protein; 10% Pro, 12% Ser, 13% Thr; Thr-rich motif from ~aa180-240 | Lotgi1\|239339 | 64.8 | 1.1 | 0.0103 | P* |
| Uncharacterized protein; domain: DUF187; similar to GEPRP_LOTIA (37% identity) | Lotgi1\|174920 | 26.8 | 214.4 | 0.6431 | P* |
| Uncharacterized protein; 22% Gly, 12% Asn | Lotgi1\|239447  Lotgi1\|175200 | 2510.9 | 24.1 | 0.4673 |  |
| Uncharacterized protein; 13% Ser, 13% Val, 12% Gly | Lotgi1\|176686 | 3.6 | 9.0 | 0.0048 |  |
| Uncharacterized protein; aa138-199 73% identity to aa49-110 of EFCB2_LOTGI | Lotgi1\|239519 | 2.2**^3^** | - | 0.0010 |  |
| Uncharacterized protein; domains: IG-like | Lotgi1\|239573 | - | 0.3 | <0.0001 |  |
| Uncharacterized protein/LUSP-20; domains: chitin_binding CBM_14/ peritrophin A (Lotgi1\|175684) | Lotgi1\|239574 | 62.9 | 30.6 | 0.1007 |  |
| Similar to histone H2B/H4 | Lotgi1\|175997  Lotgi1\|180742 | 3.9 | 2.5 | 0.0035 |  |
| Similar to embryocarcinomic antigen-related cell adhesion molecule (secreted CEACAM1a-4C1); domains: IG_like | Lotgi1\|176496 | 1.2 | 1.8 | 0.0036 |  |
| Similar to CD109-like/thioester-containing protein/α2-macroglobulin | Lotgi1\|211451  Lotgi1\|211452 | - | 0.3 | 0.0001 |  |
| Similar to follistatin-like protein; domains: EFh, Kazal_FS | Lotgi1\|112867  Lotgi1\|213075 | 1.9 | - | 0.0023 |  |
| Uncharacterized protein; 17% Cys; domains: antistasin | Lotgi1\|171918  Lotgi1\|237912 | 5.1 | - | 0.0014 |  |
| Uncharacterized protein; domain: M1_APN_2 (membrane aminopeptidase) | Lotgi1\|140786  Lotgi1\|225855 | 9.0 | 315.2 | 0.6130 |  |
| Similar to chorionic proteinase inhibitor/perlwapin/WAP; aa1-125 99.6% identity to B3A0S1 | Lotgi1\|201802  Lotgi1\|239121  PWAPL_LOTGI | nd | nd | 0.3863 |  |
| Uncharacterized protein; domains: antistasin, WAP | Lotgi1\|239125  Lotgi1\|226725 | 482.3 | 144.5 | 0.6593 |  |
| Uncharacterized protein USP8/B3A0Q4 | Lotgi1\|228268  USP8_LOTGI | 5335.7 | 9.0 | 1.2204 | P* |
| Uncharacterized protein; domain: zinc finger, CCCH-type; 17% Arg, 11% Ser, 10% Asp; all peptides from C-terminal ~150 aa | Lotgi1\|229978 | 0.2 | - | 0.0001 |  |
| Uncharacterized protein;13% Pro, 12% Ser | Lotgi1\|158905  Lotgi1\|231268 | 57.8 | - | 0.0161 |  |
| Uncharacterized protein; domains: EGF | Lotgi1\|232718 | 1.9 | - | 0.0006 |  |
| Uncharacterized shell protein 8/B3A0R3 | Lotgi1\|234885  USP13_LOTGI | >10,000 | 30.6 | 2.1302 |  |
| Uncharacterized protein; domain: otoanchorin; 12% Asp, 12% Leu, pI 4.7 | Lotgi1\|235120 | 6.4 | 17.0 | 0.0371 |  |
| EGF-like domain-containing protein 1/ B3A0R6 | Lotgi1\|235548  ELDP1_LOTIA | 3.1 | 9.0 | 0.2744 |  |
| Uncharacterized protein/ disintegrin and metalloproteinase with thrombospondin motifs;domains: ADAM_MEPRO | Lotgi1\|236770 | 12.1 | 37.7 | 0.0706 |  |
| Uncharacterized shell protein 26/B3A0P4/ BMSP-like | Lotgi1\|238526  USP26_LOTGI | 76.4 | 3261.2 | 1.4240 | P* |
| Uncharacterized protein; 14% Pro | Lotgi1\|173199  Lotgi1\|238562 | 0.4 | - | 0.0007 |  |
| Similar to 15kDa selenoprotein; domain: thioredoxin/Sep15/SelM | Lotgi1\|78136 | nd | nd | 0.0008 |  |
| Similar to palmitoyl-protein thioesterase | Lotgi1\|186317  Lotgi1\|65548 | 12.9 | 3.4 | 0.0091 |  |
| Similar to plancitoxin/DNAse II | Lotgi1\|193211  Lotgi1\|73909 | 0.8 | 1.2 | 0.0011 |  |
| Coiled-coil domain-containing protein 1/B3A0Q3 | Lotgi1\|233420  CCD1_LOTGI | 630.0 | >10,000 | 3.4915 | P* |
| Similar to adenosylhomocysteinase | Lotgi\|105848 | - | 0.5 | nd |  |
| Uncharacterized protein; domain: Dyp-type peroxidase | Lotgi1\|114599 | 0.3 | - | nd |  |
| Similar to legumain; domain:peptidase C13/legumain | Lotgi1\|115714 | - | 0.6 | nd |  |
| Similar to ferritin | Lotgi1\|123691 | - | 0.8 | nd |  |
| Similar to histone H3 | Lotgi1\|176498  etc | - | 2.2 | nd |  |
| Similar to acyl-protein thioesterase; domain: abhydrolase_3/Lysophospholipase_related | Lotgi1\|152136 | 0.6 | - | nd |  |
| Similar to heparan sulfate 2-O-sulfotransferase | Lotgi1\|175663 | - | 0.8 | nd |  |
| Similar to mitochondrial glutamate dehydrogenase | Lotgi1\|177468 | - | 0.3 | nd |  |
| Similar to H^+^-transporting ATP synthase subunit | Lotgi1\|183079 | - | 0.4 | nd |  |
| Similar to ribosomal protein S8 | Lotgi1\|186221 | - | 0.8 | nd |  |
| Similar to phosphoglycerate mutase | Lotgi1\|187919 | - | 0.4 | nd |  |
| Similar to proteasome subunit α-type | Lotgi1\|189380 | - | 0.6 | nd |  |
| Uncharacterized protein; domains: P67PHOX, SH2 motif | Lotgi1\|196407 | - | 0.8 | nd |  |
| Similar to Ras-related GTPase | Lotgi1\|198201 | - | 0.6 | nd |  |
| Similar to dynein light chain, type 1 | Lotgi1\|203845 | - | 1.5 | nd |  |
| Similar to cathepsin A; domains: carboxypeptidase/peptidase_S10 | Lotgi1\|206131 | - | 2.9 | nd |  |
| Similar to Na^+^/K^+^ ATPase, β-subunit | Lotgi1\|213719 | - | 1.5 | nd |  |
| Similar to α-fucosidase | Lotgi1\|217885 | - | 0.4 | nd |  |
| Similar to lecithin:cholesterol acyltransferase/1-O-acylceramide synthase | Lotgi1\|220528 | 0.5 | - | nd |  |
| Similar to Rab 5 | Lotgi1\|222012 | - | 1.5 | nd |  |
| Uncharacterized protein; domains: tetraspanin | Lotgi1\|228639 | - | 0.7 | nd |  |
| Uncharacterized protein; domains: cadherin repeats | Lotgi1\|229249 | - | 0.1 | nd |  |
| Similar to actin-binding protein/coactosin; domains:cofilin/tropomyosin type | Lotgi1\|231819 | - | 0.3 | nd |  |
| Similar to ADP-ribosylation factor | Lotgi1\|236204 | - | 0.7 | nd |  |
| Similar to UDP-N-acetylglucosamine pyrophosphorylase-like | Lotgi1\|233335 | 2.2 | - | nd |  |
| Uncharacterized protein | Lotgi1\|239621 | - | 1.0 | nd |  |
|  |  |  |  |  |  |

**^1^**, previously determined [17] for matrices isolated after cleaning method B; without phosphopeptide enrichment. **^2^**, MaxQuant results for combined acid-soluble and acid-insoluble fractions. **^3^**, identified only with method A or C previously [17]. P, accepted phosphopeptid(s); P*, confirmed with phosphopeptide enriched fractions; (P), not confirmed with phosphopeptide-enriched fractions; P, only in phosphopeptide-enriched fractions. N, N-glycosylation detected. AllModels accession codes are given in red. Proteins with a rounded percentage of more than 1% are shaded orange. Proteins with a rounded percentage of more than 0.1% but smaller than 1% ore shaded yellow. The MaxQuant search with a combination of raw-files from acid-soluble and acid-insoluble samples (kept apart previously), while using the same FDR, PEP and score settings as before, resulted in the loss of some peptides and corresponding trace proteins that were not identified or accepted any more. These are the entries without iBAQ percentage listed at the end of the table.
