# Supplementary material for: The Lottia gigantea shell matrix proteome: re-analysis including MaxQuant iBAQ quantitation and phosphoproteome analysis
Source: Proteome Sci. 2014 May 18;12:28. doi: 10.1186/1477-5956-12-28 (PMC4094399; doi:10.1186/1477-5956-12-28)
Supplement: Additional file 4: Table S4 — List of identified and accepted phosphopeptides and phosphoproteins from the general proteomic survey and from analysis of phosphopeptide-enriched samples. [file 1477-5956-12-28-S4.docx]

**Table S4**

**Phosphoproteins and phosphopeptides of the *Lottia gigantea* shell matrix**

|  |  |  |  |  |  |
| --- | --- | --- | --- | --- | --- |
| **Lotgi**  **accession** | **Protein name or**  **UniProt accession** | **Peptide** | **No. of P** | **Mod/**  **unmod** | **Best loc.**  **prob.** |
|  |  |  |  |  |  |
| 95414 | Similar to acidic ribosomal protein P0 (K1QWX2 _CRAGI) | _298_KEESEE**pS**DDDMGFGLFD  _298_KEE**pS**EESDDDMGFGLFD | 1 | 29/35**^2^**  3/35**^2^** | 0.99  0.99 |
| **154020** | **Uncharacterized protein** | _181_AFGGED**pS**EEMSENPTLEEISTIVK | 1 | 13/19^1^  44/65**^2^** | 1.00 |
|  |  | _218_GFFD**pS**EEMPYDSELDNNDVVIR | 1 | 17/20^1^  10/15^2^ | 1.00 |
|  |  | _95_VGDLVEPLTEAEESEA**pS**IEILK | 1 | 19/45^1^  64/98**^2^** | 1.00 |
|  |  | _95_VGDLVEPLTEAEE**pS**EASIEILK | 1 | 2/98**^2^** | 0.90 |
| **213813**  **231311** | **GEPRP_LOTGI**  **(B3A0P5)** | _34/63_LTLDIGLGNGDVERE**pS**EEAEGEGTDGR | 1 | 57/57^1^  46/93**^2^** | 1.00 |
| *232714* | *Uncharacterized protein* | _433_LELPIESHD**pS**CENSNYVK | 1 | 5/7^1^ | 1.00 |
| **162078** | **DGLSP_LOTIA**  **(B3A0P1)** | _1841_APKPSIG**pS**GLLPGLDLSSILGK  _1841_APKPSIGSGLLPGLDLS**pS**ILGK  _1841_APKP**p[SIGS]**GLLPGLDLSSILGK | 1  1  1 | 1/24**^2^**  1/24**^2^**  8/24^2^ | 0.81  0.85 |
|  |  | _1472_EYKQE**pS**PEVER | 1 | 1/142**^1^**  36/89**^2^** | 1.00 |
|  |  | _1026_FSDDG**pS**LAYGAQDNGKPGSGSPR | 1 | 2/116**^1^**  8/44**^2^** | 1.00 |
|  |  | _1501_ILITSLT**p[SS]**R | 1 | 9/32**^2^** |  |
|  |  | _169_KSGY**p[SPSVYY]**DDDDDDDDFDDDLEDR | 1 | 18/47**^2^** |  |
|  |  | _1844_PSIG**pS**GLLPGLDLSSILGK | 1 | 8/27**^2^** | 1.00 |
|  |  | _1475_QE**pS**PEVER | 1 | 5/26**^2^** | 1.00 |
|  |  | _170_SGY**p[SPSVYY]**DDDDDDDDFDDDLEDR | 1 | 24/83**^2^** |  |
|  |  | _999_YTF**pS**DDGSLAYK | 1 | 32/102**^2^** | 1.00 |
|  |  | _999_YTFSDDG**pS**LAYK | 1 | 3/869**^1^**  9/102**^2^** | 1.00 |
|  |  | _999_**pY**TFSDDGSLAYK | 1 | 8/102**^2^** | 1.00 |
| **232817** | **PLSP2_LOTGI**  **(B3A0P3)** | _261_ETPV**pS**SQPDDSISGLMGK  _261_ETPVS**pS**QPDDSISGLMGK  _261_ETPVSSQPDDSI**pS**GLMGK  _261_ETPV**p[SS]**QPDDSISGLMGK | 1  1  1  1 | 5/457**^1^**  22/291**^2^**  44/291^2^  2/291**^2^**  1/457**^1^**  26/291**^2^** | 1.00  0.90  0.93 |
|  |  | _1159_FFDAE**pS**AGPSMK | 1 | 24/46**^2^** | 1.00 |
|  |  | _567_QPDTYSPVG**pS**QPER | 1 | 5/76**^2^** | 1.00 |
|  |  | _1143_V**pS**QSESVASLSGIMPR  _1143_VSQ**pS**ESVASLSGIMPR  _1143_VSQSE**pS**VASLSGIMPR  _1143_VSQSESVA**pS**LSGIMPR  _1143_VSQSESVASL**pS**GIMPR  _1143_V**p[SQSESVASLS]**GIMPR | 1  1  1  1  1  1 | 4/46**^2^**  10/46**^2^**  7/46**^2^**  5/46^2^  3/46**^2^**  7/46**^2^** | 0.93  1  0.97  0.97  0.97 |
| 233408 | Similar to cofilin /actin-depolymer-izing factor | _2_acS**pS**GIEFPK | 1 | 7/7**^2^** | 0.87 |
| **166131** | **Similar to super-oxide dismutase** | _136_VS**p[SS]**QIETISQILNVPR  _136_V**p[SSS]**QIETISQILNVPR | 1  1 | 7/211**^1^**  6/211**^1^**  12/14**^2^** |  |
| *235497* | *PRP1_LOTGI*  *(B3A0Q1)* | _206_**pT**WYHRPAPTYAAPTK | 1 | 2/316**^1^** | 0.94 |
| **171084** | **Uncharacterized protein** | _232_**p[SS]**EVPVSDWVNYIK | 1 | 2/20**^1^**  2/2**^2^** |  |
| **238358** | **ASRP_LOTGI**  **(B3A0S2)** | _168_ALVEALD**pS**NSIDNAEDVADDIADK  _168_ALVEALDSN**pS**IDNAEDVADDIADK  _168_ALVEALD**p[SNS]**IDNAEDVADDIADK | 1  1  1 | 15/79**^2^**  1/79**^2^**  22/79**^2^** | 1.00  0.81 |
|  |  | _168_ALVEALD**pS**NSIDNAEDVADDIADKVDDINNAVK  _168_ALVEALD**p[SNS]**IDNAEDVADDIADKVDDINNAVK | 1  1 | 2/9**^1^**  5/36**^2^**  14/36**^2^** | 0.99 |
| *174205* | *Uncharacterized protein* | _551_**pS**KEVTSLPLFQTQR  _551_**p[SKEVT]**SLPLFQTQR | 1  1 | 5/15**^1^**  2/15**^1^** | 0.97 |
| **239129** | **Uncharacterized protein/LUSP-14** | _778_PVDFNNVDPFTLMNMGGMGGMGGMGGMGA  M**pS**PFGFGTEPLLK  _778_PVDFNNVDPF**p[TLMNMGGMGGMGGMGGMGA**  **MS]**PFGFGTEPLLK | 1  1 | 2/4**^1^**  3/8**^2^**  5/8**^2^** | 0.98  0.86  0.86 |
| 174652 | Uncharacterized protein | _533_FYSAPTVD**pS**QQQDTGSTPK | 1 | 2/3**^2^** | 0.92 |
| *239214* | *GSP1_LOTGI*  *(B3A0P6)* | _308_VSGTGGSSQGGSQSSSSS**p[SMMTQQMMNS]**MFGGR | 1 | 26/500**^1^** |  |
|  |  | _221_V**pT**LPFNPLILKWTK | 1 | 3/34**^1^** | 1.00 |
| **239339** | **Uncharacterized protein** | _548_DGQ**pS**IETTTPK | 1 | 1/21**^1^**  3/3**^2^** | 1.00 |
| **174920** | **Uncharacterized /similar to** | _88_MGVND**pY**DGGYQER | 1 | 1/55**^1^**  4/5**^2^** | 1.00 |
|  | **GEPRP_LOTIA** | _75_NDVEYDDD**pS**VGER  _75_NDVE**pY**DDD**pS**VGER | 1  2 | 6/22**^1^**  5/31**^2^** | 1.00  1.00 |
|  |  | _105_WEGNVE**pS**GEEGER | 1 | 1/1**^1^**  2/2**^2^** | 1.00 |
| **228268** | **USP8_LOTGI**  **(B3A0Q4)** | _171_TVGGITVDGSDV**pS**YA | 1 | 2/39**^1^**  1/1**^2^** | 1.00 |
|  |  | _171_TVGGI**pT**VDGSDVSYA | 1 | 1/39**^1^** | 1.00 |
| **238526** | **USP26_LOTGI**  **(B3A0P4)** | _450_EI**p[SGGQT]**GPTKPVVNQPR  _450_EISGGQ**pT**GPTKPVVNQPR | 1  1 | 1/213**^1^**  1/10**^2^** | 0.94 |
|  |  | _664_IIW**p[SS]**GNQQWTIAKPDGTVIK | 1 | 2/113**^1^** |  |
| **233420** | **CCD1_LOTGI**  **(B3A0Q3)** | _156_DSNDDKNDDE**pT**SDIIDDILDIVEDAK  _156_DSNDDKNDDET**pS**DIIDDILDIVEDAK  _156_DSNDDKNDDE**p[TS]**DIIDDILDIVEDAK | 1  1  1 | 1/76**^1^**  32/76**^1^**  16/37**^2^**  20/76**^1^**  12/37**^2^** | 0.79  0.98 |
|  |  | _54_ID**pS**KLDSILSELQK  _54_IDSKLD**pS**ILSELQK  _54_ID**pS**KLD**pS**ILSELQK | 1  1  2 | 9/75**^1^**  15/22**^2^**  5/75**^1^**  5/75**^1^** | 1.00  1.00  1.00 |
|  |  | _58_LD**pS**ILSELQK  _58_LDSIL**pS**ELQK | 1  1 | 5/279**^1^**  29/67**^2^**  13/279**^1^**  16/67**^2^** | 1.00  1.00 |
|  |  | _162_NDDE**pT**SDIIDDILDIVEDAK  _162_NDDET**pS**DIIDDILDIVEDAK  _162_NDDE**p[TS]**DIIDDILDIVEDAK | 1  1  1 | 1/17**^1^**  7/17**^1^**  5/15**^2^**  7/17**^1^**  4/15**^2^** | 0.86  0.82  0.92 |
|  |  | _194_VVESL**pS**KPGAVAEDDKESK  _194_VVESLSKPGAVAEDDKE**pS**K | 1  1 | 4/71**^2^**  3/184**^1^**  5/71**^2^** | 0.94  1.00 |

Entries in **bold** print indicate phosphorylation detected in both, the general survey and the analysis of phosphopeptide-enriched fractions. Entries in *italics* indicate phosphorylation detected only in the general survey. The other entries were phosphoproteins only detected in phosphopeptide-enriched fractions. **^1^**, modified vs non-modified copies of the peptide identified in the general survey i.e. without phosphopeptide enrichment. **^2^**, modified versus non-modified copies of the peptide identified in the phosphopeptide-enriched fractions. In some cases the localization was equivocal and the modified sequence is shown in square brackets. Proteins belonging to the major proteins with an estimated percentage of <0.1% of the total identified proteome are color-coded: red, >1%, orange, between 0.1% and 0.99%.
